# The Novel Link between Gene Expression Profiles of Adult T-Cell Leukemia/Lymphoma Patients’ Peripheral Blood Lymphocytes and Ferroptosis Susceptibility

**DOI:** 10.3390/genes14112005

**Published:** 2023-10-27

**Authors:** Yu Wang, Hidekatsu Iha

**Affiliations:** 1 Department of Microbiology, Oita University Faculty of Medicine, 1-1 Idaigaoka, Hasama, Yufu 879-5593, Oita, Japan; m21d9024@oita-u.ac.jp; 2 Division of Pathophysiology, The Research Center for GLOBAL and LOCAL Infectious Diseases (RCGLID), Oita University Faculty of Medicine, 1-1 Idaigaoka, Hasama, Yufu 879-5593, Oita, Japan

**Keywords:** ferroptosis, adult T-cell leukemia/lymphoma (ATL), bioinformatic analysis, precision medicine

## Abstract

Ferroptosis, a regulated cell death dependent on iron, has garnered attention as a potential broad-spectrum anticancer approach in leukemia research. However, there has been limited ferroptosis research on ATL, an aggressive T-cell malignancy caused by HTLV-1 infection. Our study employs bioinformatic analysis, utilizing dataset GSE33615, to identify 46 ferroptosis-related DEGs and 26 autophagy-related DEGs in ATL cells. These DEGs are associated with various cellular responses, chemical stress, and iron-related pathways. Autophagy-related DEGs are linked to autophagy, apoptosis, NOD-like receptor signaling, TNF signaling, and the insulin resistance pathway. PPI network analysis revealed 10 hub genes and related biomolecules. Moreover, we predicted crucial miRNAs, transcription factors, and potential pharmacological compounds. We also screened the top 20 medications based on upregulated DEGs. In summary, our study establishes an innovative link between ATL treatment and ferroptosis, offering promising avenues for novel therapeutic strategies in ATL.

## 1. Introduction

Adult T-cell leukemia/lymphoma (ATL) is a highly aggressive malignancy caused by human T-cell leukemia virus type I (HTLV-1), with an extremely poor prognosis [1,2]. Aggressive subtypes, including acute and lymphoma types (about 60% of cases), have a median overall survival of only 8–10 months [3]. Even those initially diagnosed with indolent forms, like smoldering and chronic subtypes, often progress to aggressive disease within a year [4]. HTLV-1 infection is estimated to affect 5 to 20 million people worldwide [5], with higher prevalence in regions like southwestern Japan, the Caribbean Basin, and central Africa [1]. The morbidity of ATL among individuals with chronic HTLV-1 infection ranges from 0.7 to 7.1 cases per 1000 carrier-years [6,7,8], equating to a 3–7% lifetime risk for all HTLV-1 carriers [9]. HTLV-1 transmission typically occurs through cellular contact [10]. For instance, HTLV-1-infected lymphocytes can recruit CD4 T-cells through spontaneous secretion of leukotriene B4 [11] and selectively recruit CCR4+ CD4 T-cells via CCL22 secretion [12]. The expansion of HTLV-1-infected cells is primarily driven by the viral oncoprotein Tax, which modulates essential cellular pathways controlling cell migration, virological synapses, and intracellular protein distribution [13]. Another viral oncoprotein, HBZ, plays a multifaceted role by counteracting Tax-induced cellular effects, suppressing the host’s anti-Tax immune response, inducing HTLV-1-infected cell migration and proliferation, ultimately promoting ATL onset [13].

In 2012, the term ‘ferroptosis’ was coined to describe a form of cell death reliant on iron and driven by excessive lipid peroxidation [14]. It is characterized by abnormal iron accumulation, resulting in increased reactive oxygen species (ROS) levels, oxidative stress, lipid imbalance, lipid peroxidation, DNA damage, plasma membrane rupture, and eventual cell death [14,15]. The key factors initiating oxidative cell membrane damage in ferroptosis are iron buildup and lipid peroxidation [14], which hinge on regulating the balance between oxidative damage and antioxidant defense [15,16]. Polyunsaturated fatty acids, such as arachidonic acid and adrenic acid, are particularly susceptible to peroxidation, disrupting lipid bilayers and causing membrane damage [15]. A notable morphological feature of ferroptosis is mitochondrial ultrastructure changes, including mitochondrial shrinkage, increased membrane density, outer mitochondrial membrane rupture, reduced cristae density, and compromised membrane integrity. These changes occur alongside normal nuclear morphology with unaggregated chromatin [14,17,18,19].

Autophagy, a lysosome-dependent process, removes damaged proteins and organelles, affecting various cellular responses, including metabolic balance and inflammation [20,21]. Some evidence suggests a link between increased autophagy flux and ferroptosis in cancer cells exposed to the ferroptosis-inducing agent erastin [22]. Additionally, ferroptosis can be triggered by the degradation of ferritin, known as ferritinophagy [23]. Dysregulated ferroptosis is implicated in various pathological conditions and human diseases [15,24,25], making it a promising avenue for cancer therapeutics due to the increased iron levels and susceptibility to ferroptosis induction in cancer cells [26,27,28].

Extensive research has explored the role of ferroptosis in hematological disorders, including anemia, thrombosis, and various leukemia subtypes [29,30,31,32,33,34], such as acute myeloid leukemia (AML) [29,30,31], acute lymphoblastic leukemia (ALL) [32,34], and chronic myeloid leukemia (CML) [33]. However, there is a noticeable gap in our understanding of how ferroptosis relates to adult T-cell leukemia (ATL), primarily due to the absence of comprehensive data search and analytical strategies in this context. This gap hinders our ability to fully appreciate the significance of ferroptosis in this distinct hematological malignancy.

In this study, we aimed to analyze the GSE33615 dataset to uncover distinctive gene expression profiles and differentially expressed genes (DEGs) in ATL patients. We conducted comprehensive pathway enrichment analysis of these DEGs to reveal molecular implications. Additionally, we identified hub genes within protein–protein interaction networks, which could serve as prospective biomarkers and targets for understanding ATL’s progression, shedding light on the role of ferroptosis and autophagy in this hematological condition.

## 2. Materials and Methods

### 2.1. Data Source

In this study, we initiated our research by conducting a targeted search for the keyword ‘ATL’, in the Gene Expression Omnibus (GEO) database. We identified the dataset GSE33615 [35,36,37], which was generously contributed by Nakano K, Sato A, Uchimaru K, Utsunomiya A, Yamaguchi K, and Watanabe T. The gene expression profile GSE33615 was generated using the GPL4133 Agilent-014850 Whole Human Genome Microarray 4x44k G4112F platform (Feature Number version). This dataset comprises data from 52 individuals diagnosed with ATL and 21 healthy volunteers for comparative analysis. Total RNA samples from HTLV-1 infected CD4+ T-cell of ATL patients and from CD4+ T-cells from healthy donors were subjected to Cy-3 labeling, followed by subsequent human whole genome gene expression microarray analysis. For additional comparison analysis on ferroptosis signatures among T-cell leukemia/lymphoma patients, we also obtained the datasets of peripheral T-cell lymphomas (PTCL) and angioimmunoblastic T-cell lymphoma (AITL): GSE19069, posted by the authors of [38].

### 2.2. Sample Detection and Differential Expression Analysis

Utilizing the limma package, GSE33615 was explored to conduct differential expression analysis. A |log2 Fold Change (FC)| > 1.5 and adjusted *p*-value < 0.05 were considered as significant. Heatmaps and volcano plots were generated in R using the packages ComplexHeatmap and ggplot2 by the Xiantao website.

To further investigate the involvement of ferroptosis and autophagy in our analysis, we integrated external datasets. We obtained data on ferroptosis-related genes from the FerrDb (http://zhounan.org/ferrdb/current/operations/download.html, accessed on 8 July 2023) [39] and autophagy-related genes from the HAMdb (http://hamdb.scbdd.com/home/download/, accessed on 18 July 2023) [40]. In total, we included 564 ferroptosis-related genes and 315 autophagy-related genes, which we intersected with the GSE33615 dataset to identify DEGs associated with ferroptosis and autophagy.

### 2.3. Enrichment Analysis

We conducted a comprehensive analysis of the gene expression profile from GSE33615 using Networkanalyst 3.0 (https://www.networkanalyst.ca/NetworkAnalyst/home.xhtml, accessed on 19 September 2023) [41,42,43,44,45]. This analysis encompassed Kyoto Encyclopedia of Genes and Genome (KEGG) [46,47] and Reactome pathway analysis [48]. Furthermore, we performed Gene ontology (GO) functional enrichment analysis [49] and KEGG analysis specifically for ferroptosis-related DEGs and autophagy-related DEGs. These analyses were carried out using Metascape (https://metascape.org/gp/index.html#/main/step1, accessed on 19 September 2023) [50] and ggplot2 Package in R, with results visualized through the Xiantao website (https://www.xiantaozi.com/, accessed on 4 August 2023). Our inclusion criterion for statistical significance was set at *p* < 0.05.

### 2.4. Protein–Protein Interaction Network Analysis and the Hub Genes

To identify hub genes, we utilized STRING (version 11.5) (https://cn.string-db.org/, accessed on 9 July 2023) [51], in conjunction with Cytoscape v 3.9.1 plug-in CytoHubba, specifically employing the Maximal Clique Centrality algorithm [52]. The minimum required interaction score was set at 0.4 for significant criterion.

### 2.5. The Hub Genes and Their Interactions

The transcription factor (TF)-screened gene interaction of hub genes was analyzed by Networkanalyst 3.0 utilizing ENCODE ChIP-seq data (peak intensity signal < 500 and the predicted regulatory potential score < 1 is used). The miRNA, lncRNA, and circRNA interactions with hub genes were shown using miRWalk (http://mirwalk.umm.uni-heidelberg.de/, accessed on 19 September 2023) [53], LncRBase V.2 (http://dibresources.jcbose.ac.in/zhumur/lncrbase2/start2.php, accessed on 19 September 2023) [54], and circBase (http://www.circbase.org/, accessed on 19 September 2023) [55].

### 2.6. Potential Pharmacological Targets

For the screening of potential pharmacological small molecule compounds, we turned to the cMAP (ConnectivityMap) (https://clue.io/, accessed on 10 July 2023) [56] database. This resource encompasses data on gene expression profile changes induced by 33,609 perturbagens, allowing us to compare expression signatures. Specifically, we considered connectivity scores less than 0, as they indicate that the small molecule compounds trigger gene expression changes in the opposite direction, which may hold therapeutic potential for the disease.

## 3. Results

### 3.1. Identification of Differentially Expressed Genes of GSE33615

**Figure 1:**
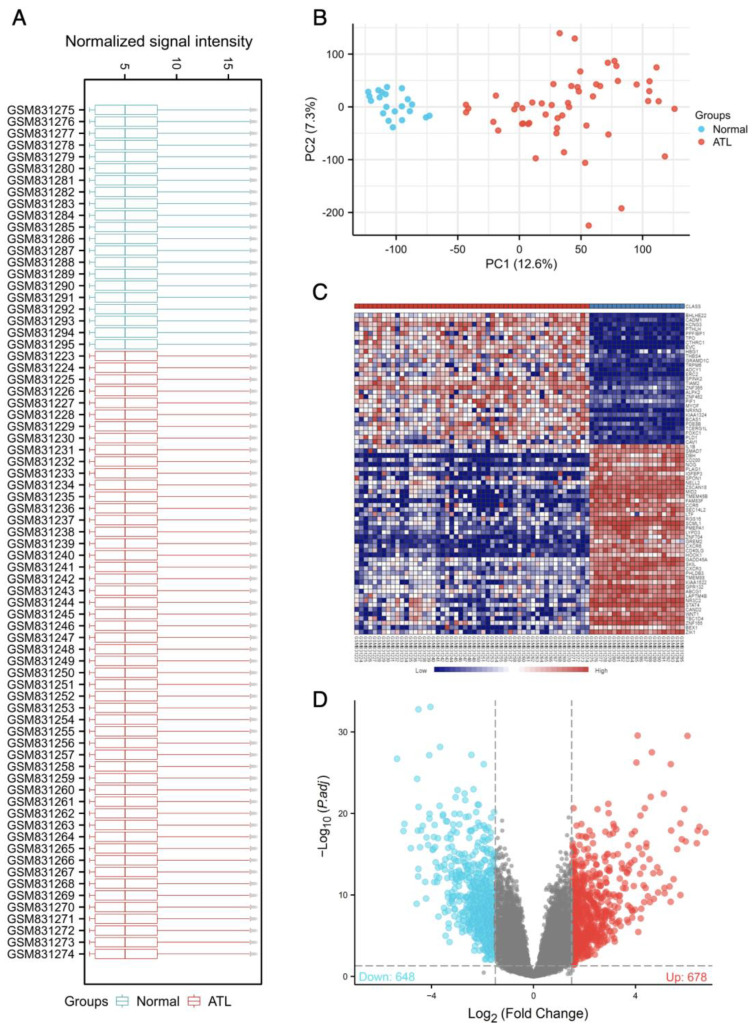
**Identification of DEGs in the ATL GEO dataset GSE33625.** (**A**) The cross−comparability evaluation of microarray data. (**B**) The gene cluster by PCA loading score. (**C**) The heat map of the dataset. (**D**) Volcanic plots of gene expression of ATL in GSE33615. 678 up-regulated genes (red dots) and 648 down-regulated genes (blue dots) were identified with a *p*-value < 0.05. Gray dots represent genes that are not statistically significant.

The gene dataset GSE33615 comprised a total of 73 samples, including 52 ATL patients and 21 healthy volunteers. To ensure data quality and across comparability of microarray data, we employed the ‘normalizeBetweenArray’ function from the limma R package to normalize the data (Figure 1A–C). Subsequently, we explored the differentially expressed genes within GSE33615.

To assess data quality and distribution, the selected samples were processed and depicted using a boxplot (Figure 1A). This plot illustrates that the samples have been appropriately centered and exhibit a numerically standardized distribution. Furthermore, the PCA plot (Figure 1B) effectively demonstrates the expected biological clustering of ATL and normal samples. It showcases the clear distinction between these two groups, highlighting their inherent biological differences. The heat map (Figure 1C) provides a visual representation of the distinguishing features between ATL and normal samples, offering insights into the gene expression patterns that set them apart.

Our analysis eventually identified 1326 genes that exhibited differential expression in ATL patients compared to healthy volunteers (with an adjusted *p*-value < 0.05 and |log2FC| > 1.5). Among these genes, 678 were upregulated and 648 were downregulated, as visualized in the volcano plot (Figure 1D).

### 3.2. The GSEA Analysis of Differentially Expressed Genes of GSE33615

**Figure 2:**
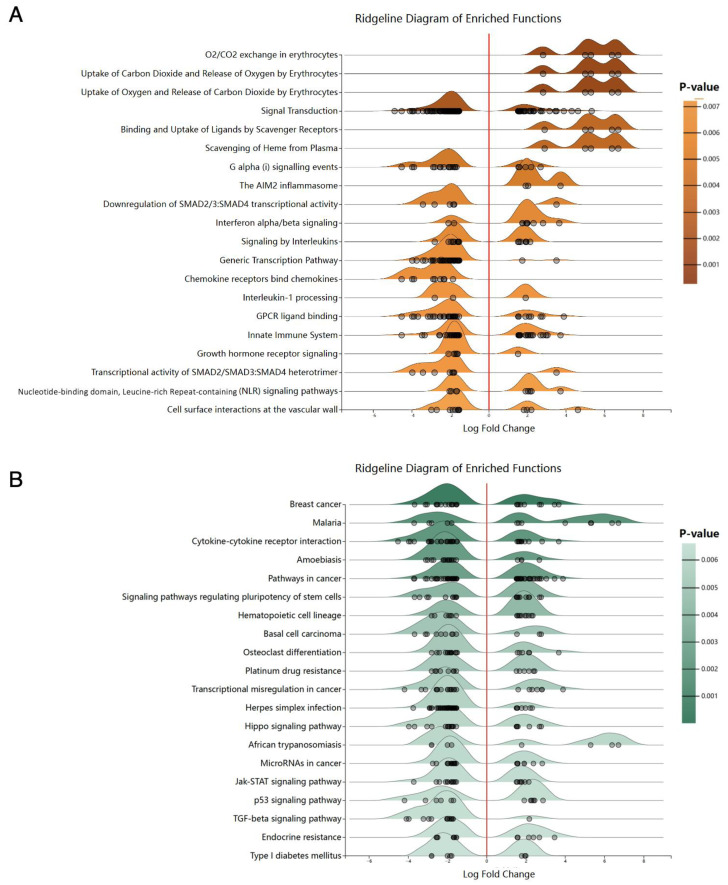
The pathway enrichment analysis of ATL whole gene expression profile by dataset GSE336156. (**A**) The Reactome analysis of GSE33615. (**B**) The KEGG analysis of GSE33615.

To identify the most enriched genes sets among all detected genes in ATL patients, we utilized the Networkanalyst website for comprehensive functional analysis. The Reactome pathway analysis (Figure 2A) revealed that the unregulated genes were notably enriched in pathways related to O_2_/CO_2_ exchange in erythrocytes, Uptake of Carbon Dioxide and Release of Oxygen by Erythrocytes, Uptake of Oxygen and Release of Carbon Dioxide by Erythrocytes, Binding and Uptake of Ligands by Scavenger Receptors, as well as Scavenging of Heme from the Plasma pathway.

The downregulated genes were enriched in several pathways, including signal transduction, G alpha (i) signaling events, downregulation of SMAD2/3: SMAD4 transcriptional activity, and the chemokine receptors binding to chemokines pathway. In addition, our KEGG analysis (Figure 2B) identified several top pathways enriched among these genes. We took particular interest in several pathways, including statistically significant ones like breast cancer, malaria, cytokine-cytokine receptor interaction, and microRNAs in cancer. Additionally, we focused on Jak-STAT, p53, and TGF-beta signaling pathways, which will each be discussed in the ‘Discussion’ section.

### 3.3. Ferroptosis-Related DEGs and Autophagy-Related DEGs

**Figure 3:**
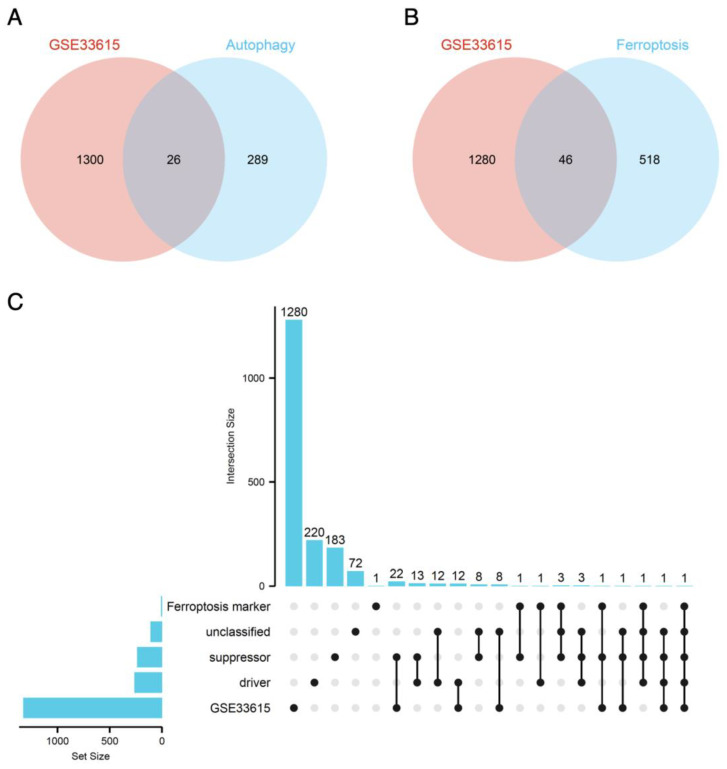
**The ferroptosis- and autophagy-related DEGs in GSE33615.** (**A**) A Venn diagram of GSE33615 DEGs and autophagy-related genes. (**B**) A Venn diagram of GSE33615 DEGs and ferroptosis-related genes. (**C**) An Upset diagram of GSE33615 DEGs along with ferroptosis marker, driver, suppressor, and unclassified.

Ferroptosis- and autophagy-associated genes were obtained from the FerrDb and HAMdb databases, respectively. Through the intersection of DEGs with these ferroptosis-associated and autophagy-associated sets, we utilized both Venn diagrams and Upset diagrams to identify 46 ferroptosis-related DEGs. Among these, 21 were upregulated and 25 were downregulated genes. Additionally, we identified 26 autophagy-related DEGs, with 9 upregulated and 17 downregulated genes in this subset of genes (Figure 3A–C, Supplementary Tables S1 and S2).

### 3.4. Functional Enrichment Analysis

The enriched GO and KEGG pathway analysis on the ferroptosis-related and autophagy-related DEGs were performed using Metascape and ggplot2 Package in R via the Xiantao website.

**Figure 4:**
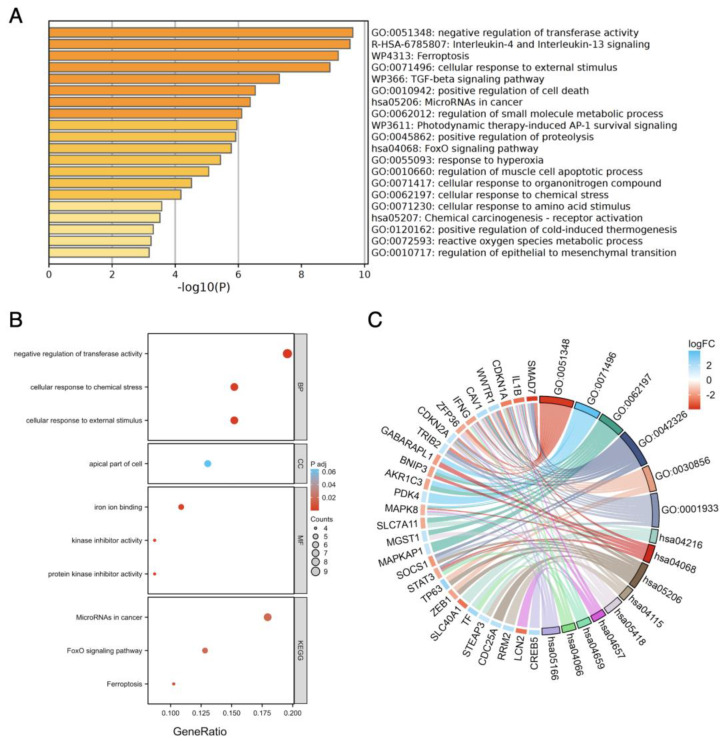
The enrichment pathway analysis of ferroptosis−related DEGs. (**A**) The enrichment pathway analysis of ferroptosis-related DEGs by Metascape. (**B**) The advanced bubble chart shows GO/KEGG enrichment significant items of ferroptosis-related DEGs via the Xiantao website. (**C**) The chord plot shows the distribution of DEGs in different GO/KEGG-enriched functions. Symbols of DEGs are shown on the left side with their fold change values mapped by color scale. Gene involvement in each term was determined by colored connecting lines.

The result of GO enrichment revealed that, in terms of biological processes (BPs), ferroptosis-related DEGs were significantly enriched in functions related to the negative regulation of transferase activity, cellular response to external stimulus, and cellular response to chemical stress. Regarding for molecular functions (MFs), ferroptosis-related DEGs exhibited significant enrichment in protein kinase inhibitor activity, kinase inhibitor activity, and iron ion binding. Furthermore, the cellular localization analysis indicated that the ferroptosis-related DEGs were predominantly located in the apical part of the cell. In the KEGG pathway-enrichment analysis, we observed significant enrichment of ferroptosis-related DEGs in various KEGG pathways, including those involved in the negative regulation of transferase activity (which includes the reduction of various transferase activities, such as methyl, acetyl, and phosphorus group transfers from donors to acceptors), interleukin-4/13 signaling (important for immune responses related to allergies and asthma), ferroptosis, and cellular responses to external stimuli (alterations in cell state or activity, including enzyme production and gene expression, triggered by external stimuli). Additionally, we focused on pathways such as the TGF-beta signaling pathway, the FoxO signaling pathway, and microRNAs in cancer due to their relevance to oncogenesis (Figure 4, Supplementary Figure S1, Supplementary Table S4).

**Figure 5:**
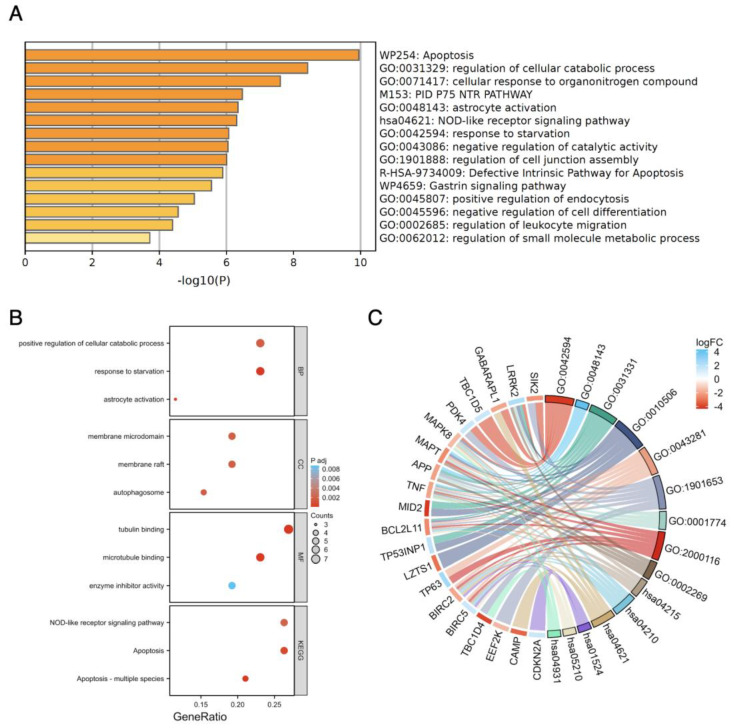
The enrichment pathway analysis of autophagy-related DEGs. (**A**) The enrichment pathway analysis of autophagy-related DEGs by Metascape. (**B**) The advanced bubble chart shows GO/KEGG enrichment significant items of autophagy-related DEGs via the Xiantao website. (**C**) The chord plot shows the distribution of DEGs in different GO/KEGG-enriched functions. Symbols of DEGs are shown on the left side with their fold change values mapped by color scale. Gene involvement in each term was determined by colored connecting lines.

For autophagy-related DEGs, the GO enrichment analysis highlighted their involvement in various BPs, including response to starvation, astrocyte activation, and positive regulation of cellular catabolic processes. These genes were primarily located in cellular components such as the autophagosome, membrane raft, and membrane microdomain. The key MFs associated with these DEGs included tubulin binding, microtubule binding, and enzyme inhibitor activity. In another KEGG pathway-enrichment analysis focusing on autophagy-related DEGs, we observed prominent enrichment in pathways related to apoptosis, the regulation of cellular catabolic processes (modulation of the rate or extent of chemical reactions and pathways leading to the breakdown of substances within individual cells), and cellular responses to organonitrogen compounds (alterations in cellular activities such as secretion, enzyme production, gene expression, etc., triggered by organonitrogen stimuli). Additionally, we identified pathways associated with immune responses and tumorigenic signals, including the PID P75 NTR PATHWAY, the NOD-like receptor signaling pathway, and the apoptosis—multiple species pathway (Figure 5, Supplementary Figure S2, Supplementary Table S5).

### 3.5. The PPI Network Analysis and Hub Gene Detection

**Figure 6:**
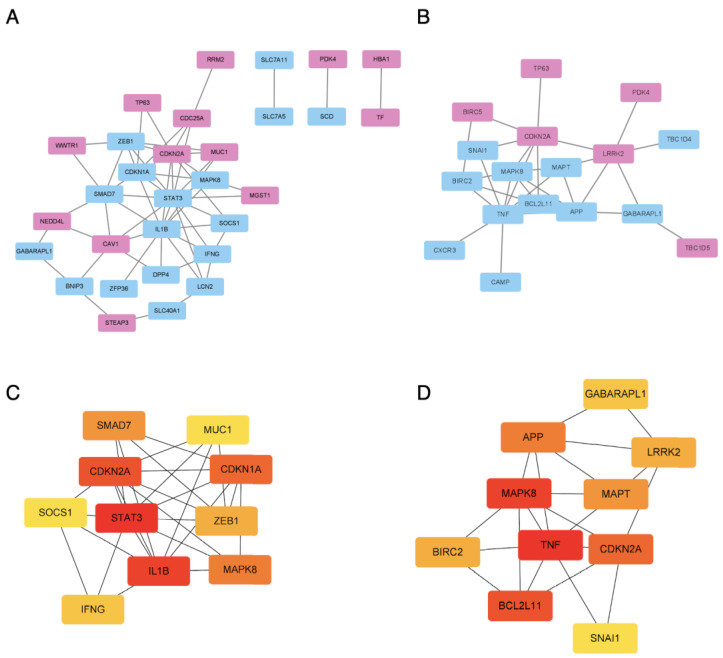
**The PPI analysis of ferroptosis-related DEGs and autophagy-related DEGs**. (**A**) The PPI analysis of ferroptosis-related DEGs; red represents upregulated genes, blue represents downregulated genes. (**B**) The PPI analysis of autophagy-related DEGs; red represents upregulated genes, blue represents downregulated genes. (**C**) The top 10 ferroptosis-related DEGs via MCC. (**D**) The top 10 autophagy-related DEGs via MCC.

To delve deeper into the role of ferroptosis- and autophagy-related DEGs, we constructed protein–protein interaction networks using STRING and Cytoscape v 3.9.1. The ferroptosis-related DEG network consisted of 30 nodes and 59 edges (Figure 6A), while the autophagy-related DEG network comprised 17 nodes and 29 edges (Figure 6C). Subsequently, we employed the MCC algorithm from the CytoHubba plugin to rank the genes within each module. This analysis identified 10 hub genes in each of the modules (Figure 6B,D).

**Figure 7:**
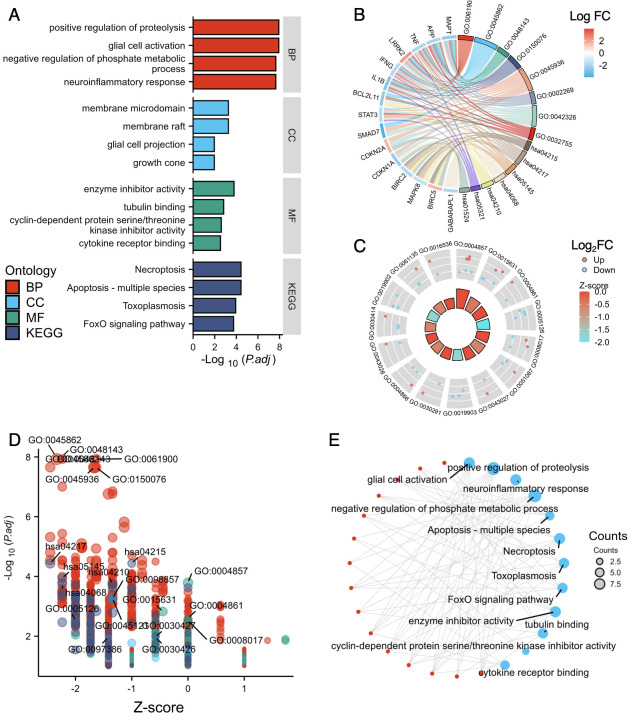
**Enrichment pathway analysis of differentially expressed genes related to ferroptosis and autophagy.** (**A**) The histogram shows the GO/KEGG enrichment analysis result of ferroptosis- and autophagy-related DEGs. (**B**) The chord plot shows the distribution of ferroptosis- and autophagy-related DEGs in different GO/KEGG-enriched functions. (**C**) The concentric circle graph displays the enrichment result data. The nodes in frame represent co-expressed gene clusters in specific biological process terms; red represents upregulated genes, blue represents downregulated genes. Each column in the inner circle corresponds to a term. The column height represents the *p*-value, with higher columns indicating smaller *p*-values. Z-scores are represented by color intensity, with negative values indicating that that rank is lower than expected. (**D**) The bubble graph illustrates the distribution of all the results obtained by enrichment. The node color represents the category corresponding to the terms. The size of node represents the amount of genes it encompasses. (**E**) The Cluego network diagram shows the relationship between the DEGs and terms; the blue nodes represent categories, red nodes represent molecules. A connection indicates that the molecule has an annotation for the corresponding categories.

The enriched GO and KEGG pathway analyses for the hub genes associated with ferroptosis and autophagy were further analyzed and visualized in Figure 7. The BPs primarily linked to these hub genes encompassed functions such as glial cell activation, positive regulation of proteolysis, neuroinflammatory response, and negative regulation of the phosphate metabolic process (Figure 7A,B,D, Supplementary Table S6).

In terms of MFs, the hub genes exhibited significant enrichment in functions such as enzyme inhibitor activity, tubulin binding, cyclin-dependent protein serine/threonine kinase inhibitor activity, and cytokine receptor binding (Figure 7A,C,D, Supplementary Table S7). Regarding KEGG pathway analysis, the hub genes displayed notable enrichment in pathways including apoptosis in multiple species, necroptosis, toxoplasmosis, and the FoxO signaling pathway (Figure 7A,B,D, Supplementary Table S6). The cellular localization analysis indicated that these hub genes were primarily located in membrane rafts, membrane microdomains, growth cones, and glial cell projections (Figure 7A,D, Supplementary Table S6). An enhanced understanding of the relationship between the DEGs and GO/KEGG terms is shown in Figure 7E.

### 3.6. Confirmation of the ATL-Specific Ferroptosis Signature

**Figure 8:**
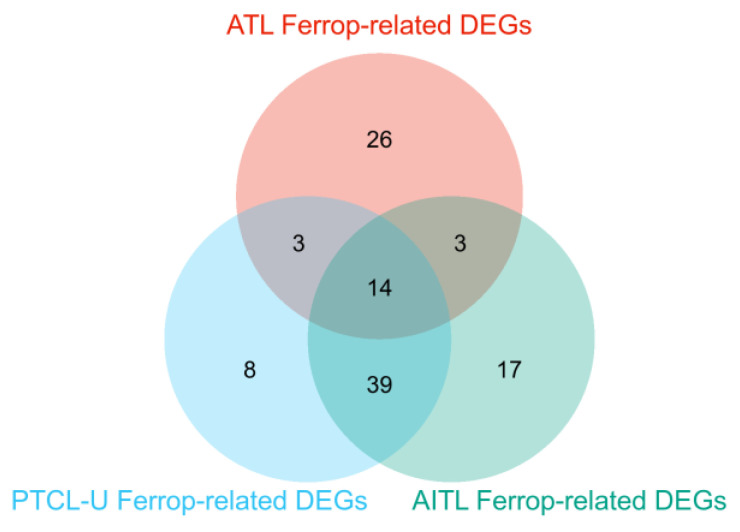
Comparisons of the ferroptosis-related DEGs among ATL, PTCL, and AITL patients PBLs. A Venn diagram of GSE33615 (ATL) and GSE19069 (PTCL and AITL) DEGs.

To validate the ATL-specific ferroptosis signature, we acquired public datasets for peripheral T-cell lymphomas (PTCLs) and angioimmunoblastic T-cell lymphoma (AITL) GSE19069 [38]. We then compared ferroptosis-associated genes among the three T-cell leukemia/lymphoma groups (Figure 8, Supplementary Tables S8 and S9). T-cell lymphomas are a diverse group with varying biological and clinical features, including ATL, which is one of 30 subtypes of mature T-cell lymphomas classified in the 2022 revised 5th edition of the World Health Organization (WHO) classification of hematolymphoid tumors [57]. Among these diseases, 14 genes were shared, including IFNG and SMAD7. However, the ferroptosis signatures in ATL patients were distinctive, with very few (three for each disease) shared genes compared to PTCL or AITL, which shared 13 times more common genes (Figure 8, Supplementary Tables S8 and S9).

### 3.7. Construction of the Target DEGs–TF Network and the Target DEGs–ncRNA Network

Out of the six upregulated hub DEGs, only four were identified as having associated transcription factors as determined by ENCODE ChIP-seq data of Networkanalyst 3.0 (Supplementary Figure S3A). Among these, MUC1 emerged as the top targeted DEG, being regulated by 68 different TFs. Additionally, BIRC5 was modulated by 30 TFs, while CDKN2A and LRRK2 were regulated by 11 and 4 TFs, respectively.

The DEGs–ncRNA interactions are represented in Supplementary Figure S1. The modules in pink and purple indicate interactions between target DEGs and circRNA or lncRNA, respectively, while modules in blue represent DEG–miRNA interactions. For a detailed view of the downregulated DEGs–miRNA interaction analysis, refer to Supplementary Figure S3B.

In this analysis, the top three target DEGs of miRNAs were identified as follows: (1) BCL2L11, which was regulated by 10 different miRNAs; (2) CDKN1A, influenced by 5 miRNAs; and (3) APP, which was modulated by 5 miRNAs (Supplementary Figure S3B).

### 3.8. Potential Pharmacology of Identified Targets DEGs

The list of upregulated DEGs was submitted to the cMAP website for a potential pharmacological analysis, where compounds were evaluated based on their connectivity scores. The top 20 potential compounds are listed in Table 1, and they include a diverse range of drug classes.

Notably, among these compounds are four EGFR inhibitors: WZ-4-145, dovitinib, canertinib, and tyrphostin-AG-1478. Additionally, there are three HDAC inhibitors: apicidin, panobinostat, and vorinostat. The list also encompasses other classes of drugs, such as calyculin (a protein phosphatase inhibitor), tricirbine (an AKT inhibitor), avraivillamide-analog-5 (a nucleophosmin inhibitor), bithionol (an autotaxin inhibitor), amsacrine (a Topoisomerase inhibitor), brefeldin-a (a protein synthesis inhibitor), as well as an HSP90 inhibitor, PKC inhibitor, glucokinase activator, and so forth.

These discoveries pave the way for promising avenues of further research and potential therapeutic directions in the diagnosing and treating of ATL.

## 4. Discussion

ATL is a complex T-cell disorder currently characterized by various clinical manifestations that result from the random proviral integration of HTLV-1. This integration is often followed by extensive genetic [58] or epigenetic [59] modifications within the host genome, further complicating the understanding and treatment of the disease. Symptoms and signs of aggressive ATL are characterized by leukemic cells displaying multi-lobulated nuclei, often referred to as ‘flower cells’. These abnormal cells can infiltrate various tissues, with a particular predilection for skin lesions [60]. Other clinical indicators of aggressive ATL include hypercalcemia and associated renal impairment, elevated serum lactate dehydrogenase (LDH) and soluble interleukin-2 receptor levels, lymphadenopathy, hepatosplenomegaly, fever, and susceptibility to opportunistic infections leading to unconsciousness [1,61]. Currently, there is an acknowledgment of various factors and components that intricately contribute to the development of ATL. However, it is important to note that the potential implications of ferroptosis in the progression of ATL have not been explored or investigated until now. This represents an intriguing and uncharted area of research that could provide valuable insights into the understanding and treatment of ATL.

In the present study, we initiated our research by conducting a comprehensive analysis of the whole gene profile within the GSE33615 dataset. In the Reactome analysis of this comprehensive gene profile, we observed that the top three enriched pathways were all associated with O_2_/CO_2_ exchange in erythrocytes. This observation is consistent with the substantial increase in energy requirements that occur following the transformation of HTLV-1-infected cells into ATL. Furthermore, our analysis suggests that ATL cells induce the glycolysis pathway, likely in response to the hypoxic microenviroment created by their rapid proliferation and infiltration into healthy tissues. This observation aligns with the notable increase in serum LDH levels observed among ATL patients, highlighting the metabolic changes that accompany the progression of this disease.

Subsequently, we proceeded to identify the DEGs related to ferroptosis and autophagy, followed by conducting enrichment analyses for each category. The functional enrichment analysis of ferroptosis-related DEGs revealed that these genes, particularly those involved in cellular response to external stimulus and chemical stress, appeared to exhibit an inhibitory effect on their respective pathways (GO:0071496, zscore = −1.8898; GO:0062197, zscore = −0.3779). These observations could potentially suggest the suppression of the ferroptosis pathway in ATL cells. This is noteworthy since acute or chronic cellular stress can precipitate cellular ferroptosis [62,63].

Iron is a crucial element that plays a pivotal role in cellular growth and homeostasis. However, an excessive accumulation of iron reserves is positively associated with an increased risk of tumor initiation and tumor growth [64,65]. Many types of cancer cells undergo a reprogramming of iron metabolism, resulting in a net influx of iron [64,65]. It is worth noting that patients diagnosed with myelodysplastic syndrome (MDS) and acute myeloid leukemia (AML) often exhibit systemic iron overload, suggesting an elevated requirement for iron by leukemic cells [65,66]. Although there is currently a lack of specific clinical investigations related to iron in ATL, these observations appear to be in line with the results of the enrichment analysis of ferroptosis-related DEGs in our study. The unexpected presence of functions related to iron ion binding and ferric iron binding within ferroptosis-related DEGs, combined with the absence of negative regulation of transferase activity such as transferrin, suggests a potential inclination of ATL cells to enhance their capability for iron uptake (GO:0005506, zscore = 0.4472; GO: 0008199, zscore = 1.4142; GO:0051348, zscore = −0.3333). However, at the same time, the insufficient response to metal ion and iron ion transport functions appears to mitigate the risk of ferroptosis to ATL cells (GO: 0006826, zscore = −0.4472; GO: 0010038, zscore = −0.3333). These findings shed light on the complex interplay of iron metabolism in ATL and its potential role in modulating susceptibility to ferroptosis. Further research is warranted to unravel the precise mechanisms underlying these observations.

From the comparison of ferroptosis signatures among other T-cell lymphomas, the ATL-specific ferroptosis signal turned out to be distinctive (Figure 8). T-cell lymphomagenesis implies the deregulation of signaling pathways, which occurs in many PTCL entities [67]. Dysregulation of the TCR pathway is a common feature of ATL, PTCL, and AITL, whereas the JAK/STAT pathway is frequently altered in PTCL with a cytotoxic immunophenotype ALK-positive or negative anaplastic large cell lymphoma (ALCL), breast-implant-associated-ALCL (Bi-ALCL), cytotoxic PTCL-NOS, and extra-nodal NK/T-cell lymphoma, nasal-type (ENKTCL). Dysregulation of the cell cycle in cancer is mostly due to inactivation of the tumor suppressor gene TP53. While alterations of TP53 and CDKN2A/PTEN have been reported in GATA3-positive PTCL-NOS, associating with complex chromosomal rearrangements and genomic instability, these alterations appear to be infrequent in ATL and AITL [68]. While PTCL and AITL shared 39 of the ferroptosis-related genes, ATL shared only 3 with both diseases (Figure 8 and Supplementary Tables S8 and S9).

The observations above suggest an intense interplay centered on the ferroptosis pathway within ATL cells. Additionally, the ferroptosis-related DEGs were also significantly enriched in processes related to the regulation of T-cell activation, Th17 cell differentiation, as well as IL-17 signaling pathway, which appeared to be related to the immunosuppressive functions exhibited by ATL cells. On a separate note, the autophagy-related DEGs also displayed patterns suggestive of immunosuppressive effects. These patterns were characterized by a partial downregulation in pathways associated with the NOD-like receptor signaling pathway, TNF signaling pathway, and leukocyte activation involved in inflammatory response. Furthermore, autophagy-related DEGs, in addition to their involvement in autophagy and apoptosis-related functions, appear to play a role in facilitating glucose uptake by ATL cells to a certain extent (hsa04931, zscore = −1.7320).

We identified 10 ferroptosis-related hub genes, with each potentially playing a distinct role in the context of ATL. These genes included six downregulated ferroptosis driver genes (IFNG, IL1B, MAPK8, SMAD7, SOCS1, ZEB1); one upregulated ferroptosis diver gene (CDKN2A); two downregulated ferroptosis suppressor genes (CDKN1A, STAT3); and one upregulated suppressor gene (MUC1). Among these genes, STAT3 is known to act as a regulator of the inflammatory response by regulating the differentiation of naive CD4+ T-cells into Th17 or Treg [69]. Interestingly, the expression of phosphorylated STAT3 has been associated with a better prognosis in ATL [70]. IL1B is a pivotal mediator of inflammatory response and is involved in T-cell activation [71]. It also promotes the differentiation of T-cells towards Th17 and synergizes with IL-12 to induce IFNG synthesis from Th1 cells [72].

In acute promyelocytic leukemia cells, IFNG exhibit a synergistic effect with As203, regulating IRF-1 expression and apoptosis induction [73]. Additionally, single nucleotide polymorphisms (SNPs) within the IFNG gene have been associated with the expansion and proliferation of hematopoietic stem cells, influencing the response to imatinib therapy in patients diagnosed with chronic myeloid leukemia (CML) [74]. MAPK8 plays a pivotal role in T-cell proliferation and differentiation, and can promote stressed-cell apoptosis by phosphorylating p53/TP53 and Yes-associated proteins YAP1 [75]. ZEB1, functioning as a transcriptional repressor, exerts negative regulation on IL-2 expression [76,77]. In the context of adult T-cell leukemia (ATL), ZEB1 functions as a tumor suppressor but is frequently disrupted through various mechanisms [78]. SOCS1 is expressed in peripheral blood T-cells in response to cytokines such as IL2, IL4, IL6, and IFNG [79]. It participates in a negative feedback loop to attenuate cytokine signaling [79,80]. Interestingly, the enforced expression of SOCS1 in lymphoid or non-lymphoid malignancies has been observed to effectively attenuate LCK-mediated cell transformation [81]. SMAD7 serves as an antagonist of TGF-β1 receptor superfamily members signaling. Overexpression of SMAD7 has been found to attenuate TGF-β-mediated inflammation and carcinogenesis [82]. However, it is worth noting that there are reported cases where the overexpression of SMAD7 is associated with an unfavorable prognosis in AML [83].

CDKN1A is a cyclin-dependent kinase inhibitor that is tightly regulated by p53. It plays a pivotal role in orchestrating p53-dependent cell cycle G1 arrest in response to various stress stimuli. CDKN2A, on the other hand, is a p53 stabilizer that represses the oncogenic effects of MDM2 by blocking MDM2-induced p53 degradation and enhancing p53-dependent transactivation. CDKN2A can also induce G2 arrest and apoptosis in a p53-independent manner. However, while both CDKN1A and CDKN2A induce cell cycle arrest, their effects in the context of ferroptosis appear to be different. It has been reported that knocking out CDKN1A enhances the sensitivity of A549 cells (lung cancer cells) to doxorubicin-induced ferroptosis, suggesting that CDKN1A inhibits ferroptosis [84]. Conversely, CDKN2A has been reported to sensitize cells to ferroptosis in a p53-independent manner. Depletion of CDK2A induces NRF2 activation and promotes cancer cell survival in response to oxidative stress [85]. However, recent studies indicate that CDKN2A-deficient gliomas exhibit heightened lipid peroxidation, leading to selective ferroptosis in the tumor [86]. Therefore, further investigation is needed to elucidate the precise role of CDKN2A in ferroptosis. MUC1 is a secreted oncogenic mucin that is abnormally expressed in ATL cells and AML blasts [87]. It is associated with an unfavorable prognosis in ATL patients [88].

Based on the 10 ferroptosis-related hub genes, we identified the top 20 compounds that may suppress ATL growth (Table 1). Among these, five small molecules (triciribine, panobinostat, vorinostat, amasacrine, and dovitinib) are currently in global phase I/II clinical trials (Supplementary Table S7). Several molecular targeting drugs for ATL treatment have been approved over the last decade. These include mogamulizumab (an anti-CCR4 monoclonal antibody) [89,90], Lenalidomide (an immunomodulatory drug) [91], Brentuximab vedotin (an anti-CD30 monoclonal antibody conjugated with an antimitotic agent, monomethyl auristatin E) [92], and Tucidinostat (an HDAC inhibitor) [93], and Valemetostat (EZH1/2 inhibitor) [94,95].

**Figure 9:**
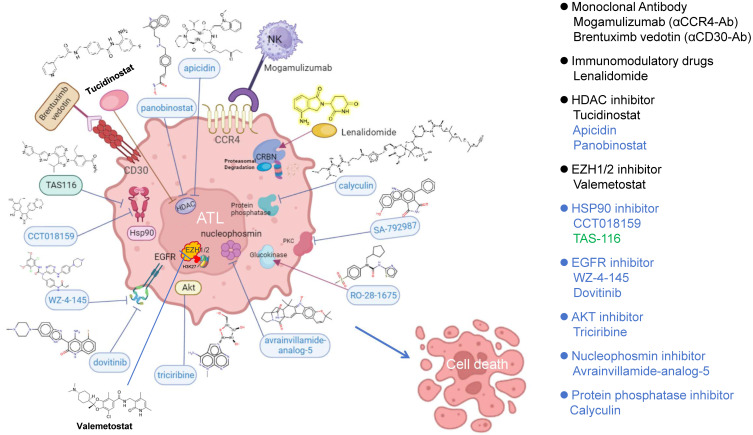
Approved and ferroptosis-inducing drug candidates for ATL therapy. Four approved molecular targeting drugs (in black), ten potential ferroptosis inducers (in blue), and a novel Hsp90 inhibitor TAS-116 (in green) examined by us [99,100,101].

In addition to these approved drugs, we have summarized 10 candidate drugs that may induce ferroptosis in ATL cells (Figure 9). These include Apicidin, a cyclic tetrapeptide with antiproliferative activity against various cancer cells, including leukemia [96]. Panobinostat, another new class of pan-HDAC inhibitor, was approved by the FDA and EMA for use in combination with bortezomib and dexamethasone for the treatment of multiple myeloma [97]. However, some research has suggested it may not be safe for elderly AML patients due to its lack of specificity [98].

HSP90 inhibitors play a crucial role in the NF-κB-mediated anti-apoptosis of ATL cells. Besides CCT018159 [102], this category includes compounds such as 17-DMAG, NVP-AUY922, and TAS-116, which have shown significant suppressive activity against ATL [99,100,101]. TAS-116 has been approved for use in gastrointestinal stromal tumor (GIST) patients [103].

Two EGFR inhibitors are also listed. WZ-4-145 has been reported as a candidate for treating pancreatic neuroendocrine tumors [104]. Dovitinib targets FGFR1/2/3 and is under clinical trial investigation as an anti-tumor drug, with FGFR1 chromosomal translocation associated with 8p11 myeloproliferative syndrome, and FGFR3 is implicated in multiple myeloma and peripheral T-cell lymphoma [105].

Triciribine, an AKT inhibitor, has been studied in phase I/II clinical trials and has shown effectiveness in reducing the risk of elevated Akt levels in patients with advanced hematological malignancies (AML, CCML, ALL, CLL) while inducing cell death through the modulation of Akt and its substrate BAD under well-tolerated conditions [106].

Avrainvillamide, a nucleophosmin inhibitor, has shown potential inhibitory effects on the abnormal trafficking of mutated NPM1, which causes acute myeloid leukemia (AML) [107].

SA-792987, a PCK inhibitor targeting Wee1 activity, has been listed as an anti-neoplastic agent against nine malignant cell lines [108].

RO-28-1675, a glucokinase activator originally developed as an allosteric activator of glucokinase for diabetes treatment [109], may induce anti-ATL activity through ferroptosis, possibly due to its role in enhancing glycolytic activity in response to hepatitis virus infection in liver cells [110].

## 5. Conclusions

In this study, we analyzed the GSE33615 dataset, allowing us to identify multiple targets associated with ferroptosis and autophagy, along with related molecules and corresponding small-molecule compounds. Our findings indicate a partial downregulation of the ferroptosis pathway in ATL cells. Leveraging the increased iron demand in cancer cells to spare normal cells and selectively target cancer cells represents a promising opportunity [111]. A recent study has demonstrated that the ferroptosis inducer erastin can overcome chemotherapy resistance and enhance the sensitivity of AML cell lines to chemotherapy treatment [112].

Furthermore, research into ferroptosis nanotherapeutic technology for hematological malignancies is also progressing, highlighting the potential of ferroptosis induction as a treatment strategy for ATL [17,113]. This study represents the pioneering investigation into the interplay between ATL and pathways associated with ferroptosis. While it sheds light on an alternative approach for ATL treatment, it is not devoid of limitation. For instance, the potential for false positives due to the enrichment method cannot be ruled out, and biases may have been introduced by sample constraints. However, both the existing and forthcoming results have the potential to aid in the identification of novel therapeutic targets. Further efforts in data collection, exploration, and the screening of related genes remain essential in the pursuit of ATL and ferroptosis-related treatment alternatives.

## Figures and Tables

**Table 1:** Top 20 predicted potential compounds from cMap for adult T-cell leukemia treatment based on the ferroptosis- and autophagy-related DEGs.

Rank	Score	ID	Name	Description
1	−99.93	BRD-A47513740	Calyculin	Protein phosphatase inhibitor
2	−99.86	BRD-A70731303	Avrainvillamide-analog-5	Nucleophosmin inhibitor
3	−99.83	BRD-U25771771	WZ-4-145	EGFR inhibitor
4	−99.44	BRD-K65503129	CCT018159	HSP90-inhibitor
5	−99.26	BRD-K80431395	Triciribine	AKT inhibitor
6	−99.15	BRD-K85402309	Dovitinib	EGFR inhibitor
7	−99.12	BRD-K64606589	Apicidin	HDAC inhibitor
8	−99.05	BRD-K02130563	Panobinostat	HDAC inhibitor
9	−99.03	BRD-K82823804	SA-792987	PKC inhibitor
10	−99.01	BRD-K21672174	RO-28-1675	Glucokinase activator
11	−98.98	BRD-K50168500	Canertinib	EGFR inhibitor
12	−98.94	BRD-K68336408	Tyrphostin-AG-1478	EGFR inhibitor
13	−98.91	BRD-K12502280	TG-101348	FLT3 inhibitor
14	−98.91	BRD-K39120595	Bithionol	Autotaxin inhibitor
15	−98.77	BRD-K98490050	Amsacrine	Topoisomerase inhibitor
16	−98.77	BRD-K81418486	Vorinostat	HDAC inhibitor
17	−98.72	BRD-A17065207	Brefeldin-a	Protein synthesis inhibitor
18	−98.70	BRD-K10705233	GW-405833	Cannabinoid receptor agonist
19	−98.64	BRD-K51575138	TPCA-1	IKK inhibitor
20	−98.61	BRD-K04853698	LDN-193189	Serine/threonine kinase inhibitor

## Data Availability

We utilized public dataset GSE33615, which was generously contributed by Nakano K, Sato A, Uchimaru K, Utsunomiya A, Yamaguchi K, and Watanabe T. Sets of genes related to ferroptosis and autophagy were obtained from the FerrDb and HAMdb databases, respectively. We extend our gratitude to the respective research teams for their efforts in creating and maintaining these resources.

## Supplementary Materials

The following supporting information can be downloaded at https://www.mdpi.com/article/10.3390/genes14112005/s1.

## Abbreviations

Adult T-cell leukemia/lymphoma (ATL); Acute lymphocytic leukemia (ALL); Acute myeloid leukemia (AML); Biological processes (BPs); Cellular component (CC); Chronic lymphocytic leukemia (CLL); Chronic myeloid leukemia (CML); Cutaneous T-cells lymphoma (CTCL); Differentially expressed genes (DEGs); Gene ontology (GO); Kyoto Encyclopedia of Genes and Genome (KEGG); Maximal Clique Centrality (MCC); Molecular functions (MFs); Myelodysplastic syndrome (MDS); Protein–protein interaction (PPI).

## References

[1] Ishitsuka K., Tamura K. (2014). Human T-cell leukaemia virus type I and adult T-cell leukaemia-lymphoma. Lancet Oncol..

[2] Uchiyama T.Y.J., Sagawa K., Takatsuki K., Uchino H., Uchiyama T., Yodoi J., Sagawa K., Takatsuki K., Uchino H. (1977). Adult T-cell leukemia: Clinical and hematologic features of 16 cases. Blood.

[3] Katsuya H., Ishitsuka K., Utsunomiya A., Hanada S., Eto T., Moriuchi Y., Saburi Y., Miyahara M., Sueoka E., Uike N. (2015). Treatment and survival among 1594 patients with ATL. Blood.

[4] Takasaki Y., Iwanaga M., Imaizumi Y., Tawara M., Joh T., Kohno T., Yamada Y., Kamihira S., Ikeda S., Miyazaki Y. (2010). Long-term study of indolent adult T-cell leukemia-lymphoma. Blood.

[5] Gessain A., Cassar O. (2012). Epidemiological Aspects and World Distribution of HTLV-1 Infection. Front. Microbiol..

[6] Arisawa K., Soda M., Akahoshi M., Fujiwara S., Uemura H., Hiyoshi M., Takeda H., Kashino W., Suyama A. (2006). Human T-cell lymphotropic virus type-1 infection and risk of cancer: 15.4 year longitudinal study among atomic bomb survivors in Nagasaki, Japan. Cancer Sci..

[7] Arisawa K., Sobue T., Yoshimi I., Soda M., Shirahama S., Doi H., Katamine S., Saito H., Urata M. (2003). Human T-lymphotropic virus type-I infection, survival and cancer risk in southwestern Japan: A prospective cohort study. Cancer Causes Control.

[8] Iwanaga M., Watanabe T., Utsunomiya A., Okayama A., Uchimaru K., Koh K.R., Ogata M., Kikuchi H., Sagara Y., Uozumi K. (2010). Human T-cell leukemia virus type I (HTLV-1) proviral load and disease progression in asymptomatic HTLV-1 carriers: A nationwide prospective study in Japan. Blood.

[9] Iwanaga M., Watanabe T., Yamaguchi K. (2012). Adult T-cell leukemia: A review of epidemiological evidence. Front. Microbiol..

[10] Pique C., Jones K.S. (2012). Pathways of cell-cell transmission of HTLV-1. Front. Microbiol..

[11] Percher F., Curis C., Pérès E., Artesi M., Rosewick N., Jeannin P., Gessain A., Gout O., Mahieux R., Ceccaldi P.E. (2017). HTLV-1-induced leukotriene B4 secretion by T cells promotes T cell recruitment and virus propagation. Nat. Commun..

[12] Hieshima K., Nagakubo D., Nakayama T., Shirakawa A.K., Jin Z., Yoshie O. (2008). Tax-inducible production of CC chemokine ligand 22 by human T cell leukemia virus type 1 (HTLV-1)-infected T cells promotes preferential transmission of HTLV-1 to CCR4-expressing CD4+ T cells. J. Immunol..

[13] El Hajj H., Bazarbachi A. (2022). Interplay between innate immunity and the viral oncoproteins Tax and HBZ in the pathogenesis and therapeutic response of HTLV-1 associated adult T cell leukemia. Front. Immunol..

[14] Dixon S.J., Lemberg K.M., Lamprecht M.R., Skouta R., Zaitsev E.M., Gleason C.E., Patel D.N., Bauer A.J., Cantley A.M., Yang W.S. (2012). Ferroptosis: An iron-dependent form of nonapoptotic cell death. Cell.

[15] Chen X., Kang R., Kroemer G., Tang D. (2021). Broadening horizons: The role of ferroptosis in cancer. Nat. Rev. Clin. Oncol..

[16] Kuang F., Liu J., Tang D., Kang R. (2020). Oxidative Damage and Antioxidant Defense in Ferroptosis. Front. Cell Dev. Biol..

[17] Kim S.E., Zhang L., Ma K., Riegman M., Chen F., Ingold I., Conrad M., Turker M.Z., Gao M., Jiang X. (2016). Ultrasmall nanoparticles induce ferroptosis in nutrient-deprived cancer cells and suppress tumour growth. Nat. Nanotechnol..

[18] Doll S., Proneth B., Tyurina Y.Y., Panzilius E., Kobayashi S., Ingold I., Irmler M., Beckers J., Aichler M., Walch A. (2017). ACSL4 dictates ferroptosis sensitivity by shaping cellular lipid composition. Nat. Chem. Biol..

[19] Doll S., Conrad M. (2017). Iron and ferroptosis: A still ill-defined liaison. IUBMB Life.

[20] Mizushima N., Levine B. (2010). Autophagy in mammalian development and differentiation. Nat. Cell Biol..

[21] Klionsky D.J., Emr S.D. (2000). Autophagy as a regulated pathway of cellular degradation. Science.

[22] Hou W., Xie Y., Song X., Sun X., Lotze M.T., Zeh H.J., Kang R., Tang D. (2016). Autophagy promotes ferroptosis by degradation of ferritin. Autophagy.

[23] Ajoolabady A., Aslkhodapasandhokmabad H., Libby P., Tuomilehto J., Lip G.Y.H., Penninger J.M., Richardson D.R., Tang D., Zhou H., Wang S. (2021). Ferritinophagy and ferroptosis in the management of metabolic diseases. Trends Endocrinol. Metab..

[24] Friedmann Angeli J.P., Schneider M., Proneth B., Tyurina Y.Y., Tyurin V.A., Hammond V.J., Herbach N., Aichler M., Walch A., Eggenhofer E. (2014). Inactivation of the ferroptosis regulator Gpx4 triggers acute renal failure in mice. Nat. Cell Biol..

[25] Chen Z., Jiang J., Fu N., Chen L. (2022). Targetting ferroptosis for blood cell-related diseases. J. Drug Target..

[26] Tang R., Xu J., Zhang B., Liu J., Liang C., Hua J., Meng Q., Yu X., Shi S. (2020). Ferroptosis, necroptosis, and pyroptosis in anticancer immunity. J. Hematol. Oncol..

[27] Beretta G.L., Zaffaroni N. (2023). Radiotherapy-induced ferroptosis for cancer treatment. Front. Mol. Biosci..

[28] Lee J., Roh J.L. (2023). Unleashing Ferroptosis in Human Cancers: Targeting Ferroptosis Suppressor Protein 1 for Overcoming Therapy Resistance. Antioxidants.

[29] Greco G., Schnekenburger M., Catanzaro E., Turrini E., Ferrini F., Sestili P., Diederich M., Fimognari C. (2021). Discovery of Sulforaphane as an Inducer of Ferroptosis in U-937 Leukemia Cells: Expanding Its Anticancer Potential. Cancers.

[30] Wei J., Nai G.Y., Dai Y., Huang X.J., Xiong M.Y., Yao X.Y., Huang Z.N., Li S.N., Zhou W.J., Huang Y. (2021). Dipetidyl peptidase-4 and transferrin receptor serve as prognostic biomarkers for acute myeloid leukemia. Ann. Transl. Med..

[31] Yusuf R.Z., Saez B., Sharda A., van Gastel N., Yu V.W.C., Baryawno N., Scadden E.W., Acharya S., Chattophadhyay S., Huang C. (2020). Aldehyde dehydrogenase 3a2 protects AML cells from oxidative death and the synthetic lethality of ferroptosis inducers. Blood.

[32] Jin L., Tong L. (2021). PAQR3 inhibits proliferation and aggravates ferroptosis in acute lymphoblastic leukemia through modulation Nrf2 stability. Immun. Inflamm. Dis..

[33] Liu S., Wu W., Chen Q., Zheng Z., Jiang X., Xue Y., Lin D. (2021). TXNRD1: A Key Regulator Involved in the Ferroptosis of CML Cells Induced by Cysteine Depletion In Vitro. Oxid. Med. Cell. Longev..

[34] Pontel L.B., Bueno-Costa A., Morellato A.E., Carvalho Santos J., Roue G., Esteller M. (2022). Acute lymphoblastic leukemia necessitates GSH-dependent ferroptosis defenses to overcome FSP1-epigenetic silencing. Redox Biol..

[35] Yamagishi M., Nakano K., Miyake A., Yamochi T., Kagami Y., Tsutsumi A., Matsuda Y., Sato-Otsubo A., Muto S., Utsunomiya A. (2012). Polycomb-mediated loss of miR-31 activates NIK-dependent NF-kappaB pathway in adult T cell leukemia and other cancers. Cancer Cell.

[36] Fujikawa D., Nakagawa S., Hori M., Kurokawa N., Soejima A., Nakano K., Yamochi T., Nakashima M., Kobayashi S., Tanaka Y. (2016). Polycomb-dependent epigenetic landscape in adult T-cell leukemia. Blood.

[37] Yamagishi M., Kubokawa M., Kuze Y., Suzuki A., Yokomizo A., Kobayashi S., Nakashima M., Makiyama J., Iwanaga M., Fukuda T. (2021). Chronological genome and single-cell transcriptome integration characterizes the evolutionary process of adult T cell leukemia-lymphoma. Nat. Commun..

[38] Iqbal J., Weisenburger D.D., Greiner T.C., Vose J.M., McKeithan T., Kucuk C., Geng H., Deffenbacher K., Smith L., Dybkaer K. (2010). Molecular signatures to improve diagnosis in peripheral T-cell lymphoma and prognostication in angioimmunoblastic T-cell lymphoma. Blood.

[39] Zhou N., Yuan X., Du Q., Zhang Z., Shi X., Bao J., Ning Y., Peng L. (2023). FerrDb V2: Update of the manually curated database of ferroptosis regulators and ferroptosis-disease associations. Nucleic Acids Res..

[40] Wang N.N., Dong J., Zhang L., Ouyang D., Cheng Y., Chen A.F., Lu A.P., Cao D.S. (2018). HAMdb: A database of human autophagy modulators with specific pathway and disease information. J. Cheminform..

[41] Xia J., Fjell C.D., Mayer M.L., Pena O.M., Wishart D.S., Hancock R.E. (2013). INMEX—A web-based tool for integrative meta-analysis of expression data. Nucleic Acids Res..

[42] Xia J., Lyle N.H., Mayer M.L., Pena O.M., Hancock R.E. (2013). INVEX—A web-based tool for integrative visualization of expression data. Bioinformatics.

[43] Zhou G., Soufan O., Ewald J., Hancock R.E.W., Basu N., Xia J. (2019). NetworkAnalyst 3.0: A visual analytics platform for comprehensive gene expression profiling and meta-analysis. Nucleic Acids Res..

[44] Xia J., Gill E.E., Hancock R.E. (2015). NetworkAnalyst for statistical, visual and network-based meta-analysis of gene expression data. Nat. Protoc..

[45] Xia J., Benner M.J., Hancock R.E. (2014). NetworkAnalyst--integrative approaches for protein-protein interaction network analysis and visual exploration. Nucleic Acids Res..

[46] Wixon J., Kell D. (2000). The Kyoto encyclopedia of genes and genomes—KEGG. Yeast.

[47] Kanehisa M., Goto S. (2000). KEGG: Kyoto encyclopedia of genes and genomes. Nucleic Acids Res..

[48] Jassal B., Matthews L., Viteri G., Gong C., Lorente P., Fabregat A., Sidiropoulos K., Cook J., Gillespie M., Haw R. (2018). The Reactome Pathway Knowledgebase. Nucleic Acids Res..

[49] Ashburner M., Ball C.A., Blake J.A., Botstein D., Butler H., Cherry J.M., Davis A.P., Dolinski K., Dwight S.S., Eppig J.T. (2000). Gene ontology: Tool for the unification of biology. The Gene Ontology Consortium. Nat. Genet..

[50] Zhou Y., Zhou B., Pache L., Chang M., Khodabakhshi A.H., Tanaseichuk O., Benner C., Chanda S.K. (2019). Metascape provides a biologist-oriented resource for the analysis of systems-level datasets. Nat. Commun..

[51] Szklarczyk D., Kirsch R., Koutrouli M., Nastou K., Mehryary F., Hachilif R., Gable A.L., Fang T., Doncheva N.T., Pyysalo S. (2023). The STRING database in 2023: Protein-protein association networks and functional enrichment analyses for any sequenced genome of interest. Nucleic Acids Res..

[52] Shannon P., Markiel A., Ozier O., Baliga N.S., Wang J.T., Ramage D., Amin N., Schwikowski B., Ideker T. (2003). Cytoscape: A software environment for integrated models of biomolecular interaction networks. Genome Res..

[53] Sticht C., De La Torre C., Parveen A., Gretz N. (2018). miRWalk: An online resource for prediction of microRNA binding sites. PLoS ONE.

[54] Das T., Deb A., Parida S., Mondal S., Khatua S., Ghosh Z. (2021). LncRBase V.2: An updated resource for multispecies lncRNAs and ClinicLSNP hosting genetic variants in lncRNAs for cancer patients. RNA Biol..

[55] Glazar P., Papavasileiou P., Rajewsky N. (2014). circBase: A database for circular RNAs. RNA.

[56] Subramanian A., Narayan R., Corsello S.M., Peck D.D., Natoli T.E., Lu X., Gould J., Davis J.F., Tubelli A.A., Asiedu J.K. (2017). A Next Generation Connectivity Map: L1000 Platform and the First 1,000,000 Profiles. Cell.

[57] Savage K.J. (2011). Therapies for peripheral T-cell lymphomas. Hematol. Am. Soc. Hematol. Educ. Program..

[58] Kataoka K., Nagata Y., Kitanaka A., Shiraishi Y., Shimamura T., Yasunaga J., Totoki Y., Chiba K., Sato-Otsubo A., Nagae G. (2015). Integrated molecular analysis of adult T cell leukemia/lymphoma. Nat. Genet..

[59] Yamagishi M., Fujikawa D., Watanabe T., Uchimaru K. (2018). HTLV-1-Mediated Epigenetic Pathway to Adult T-Cell Leukemia-Lymphoma. Front. Microbiol..

[60] Matsuoka M., Jeang K.T. (2007). Human T-cell leukaemia virus type 1 (HTLV-1) infectivity and cellular transformation. Nat. Rev. Cancer.

[61] Shimoyama M. (1991). Diagnostic criteria and classification of clinical subtypes of adult T-cell leukaemia-lymphoma. A report from the Lymphoma Study Group (1984–87). Br. J. Haematol..

[62] Hirschhorn T., Stockwell B.R. (2019). The development of the concept of ferroptosis. Free Radic. Biol. Med..

[63] Zheng J., Conrad M. (2020). The Metabolic Underpinnings of Ferroptosis. Cell Metab..

[64] Torti S.V., Torti F.M. (2013). Iron and cancer: More ore to be mined. Nat. Rev. Cancer.

[65] Wang F., Lv H., Zhao B., Zhou L., Wang S., Luo J., Liu J., Shang P. (2019). Iron and leukemia: New insights for future treatments. J. Exp. Clin. Cancer Res..

[66] Weber S., Parmon A., Kurrle N., Schnutgen F., Serve H. (2020). The Clinical Significance of Iron Overload and Iron Metabolism in Myelodysplastic Syndrome and Acute Myeloid Leukemia. Front. Immunol..

[67] Zain J., Kallam A. (2023). Challenges in nodal peripheral T-cell lymphomas: From biological advances to clinical applicability. Front. Oncol..

[68] Drieux F., Lemonnier F., Gaulard P. (2023). How molecular advances may improve the diagnosis and management of PTCL patients. Front. Oncol..

[69] Ma L., Huang C., Wang X.J., Xin D.E., Wang L.S., Zou Q.C., Zhang Y.S., Tan M.D., Wang Y.M., Zhao T.C. (2017). Lysyl Oxidase 3 Is a Dual-Specificity Enzyme Involved in STAT3 Deacetylation and Deacetylimination Modulation. Mol. Cell.

[70] Morichika K., Karube K., Kayo H., Uchino S., Nishi Y., Nakachi S., Okamoto S., Morishima S., Ohshiro K., Nakazato I. (2019). Phosphorylated STAT3 expression predicts better prognosis in smoldering type of adult T-cell leukemia/lymphoma. Cancer Sci..

[71] Van Damme J., De Ley M., Opdenakker G., Billiau A., De Somer P., Van Beeumen J. (1985). Homogeneous interferon-inducing 22K factor is related to endogenous pyrogen and interleukin-1. Nature.

[72] Tominaga K., Yoshimoto T., Torigoe K., Kurimoto M., Matsui K., Hada T., Okamura H., Nakanishi K. (2000). IL-12 synergizes with IL-18 or IL-1beta for IFN-gamma production from human T cells. Int. Immunol..

[73] El Bougrini J., Pampin M., Chelbi-Alix M.K. (2006). Arsenic enhances the apoptosis induced by interferon gamma: Key role of IRF-1. Cell. Mol. Biol..

[74] Kim D.H., Kong J.H., Byeun J.Y., Jung C.W., Xu W., Liu X., Kamel-Reid S., Kim Y.K., Kim H.J., Lipton J.H. (2010). The IFNG (IFN-gamma) genotype predicts cytogenetic and molecular response to imatinib therapy in chronic myeloid leukemia. Clin. Cancer Res..

[75] Tomlinson V., Gudmundsdottir K., Luong P., Leung K.Y., Knebel A., Basu S. (2010). JNK phosphorylates Yes-associated protein (YAP) to regulate apoptosis. Cell Death Dis..

[76] Williams T.M., Moolten D., Burlein J., Romano J., Bhaerman R., Godillot A., Mellon M., Rauscher F.J., Kant J.A. (1991). Identification of a zinc finger protein that inhibits IL-2 gene expression. Science.

[77] Williams T.M., Montoya G., Wu Y., Eddy R.L., Byers M.G., Shows T.B. (1992). The TCF8 gene encoding a zinc finger protein (Nil-2-a) resides on human chromosome 10p11.2. Genomics.

[78] Hidaka T., Nakahata S., Hatakeyama K., Hamasaki M., Yamashita K., Kohno T., Arai Y., Taki T., Nishida K., Okayama A. (2008). Down-regulation of TCF8 is involved in the leukemogenesis of adult T-cell leukemia/lymphoma. Blood.

[79] Starr R., Willson T.A., Viney E.M., Murray L.J., Rayner J.R., Jenkins B.J., Gonda T.J., Alexander W.S., Metcalf D., Nicola N.A. (1997). A family of cytokine-inducible inhibitors of signalling. Nature.

[80] Lavens D., Ulrichts P., Catteeuw D., Gevaert K., Vandekerckhove J., Peelman F., Eyckerman S., Tavernier J. (2007). The C-terminus of CIS defines its interaction pattern. Biochem. J..

[81] Cooper J.C., Shi M., Chueh F.Y., Venkitachalam S., Yu C.L. (2010). Enforced SOCS1 and SOCS3 expression attenuates Lck-mediated cellular transformation. Int. J. Oncol..

[82] Yan X., Liu Z., Chen Y. (2009). Regulation of TGF-beta signaling by Smad7. Acta Biochim. Biophys. Sin. (Shanghai).

[83] Shehata M.M., Sallam A.M., Naguib M.G., El-Mesallamy H.O. (2021). Overexpression of BAMBI and SMAD7 impacts prognosis of acute myeloid leukemia patients: A potential TERT non-canonical role. Cancer Biomark..

[84] Koyanagi A., Kotani H., Iida Y., Tanino R., Kartika I.D., Kishimoto K., Harada M. (2022). Protective roles of cytoplasmic p21(Cip1) (/Waf1) in senolysis and ferroptosis of lung cancer cells. Cell Prolif..

[85] Chen D., Tavana O., Chu B., Erber L., Chen Y., Baer R., Gu W. (2017). NRF2 Is a Major Target of ARF in p53-Independent Tumor Suppression. Mol. Cell..

[86] Minami J.K., Morrow D., Bayley N.A., Fernandez E.G., Salinas J.J., Tse C., Zhu H., Su B., Plawat R., Jones A. (2023). CDKN2A deletion remodels lipid metabolism to prime glioblastoma for ferroptosis. Cancer Cell.

[87] Stroopinsky D., Rosenblatt J., Ito K., Mills H., Yin L., Rajabi H., Vasir B., Kufe T., Luptakova K., Arnason J. (2013). MUC1 is a potential target for the treatment of acute myeloid leukemia stem cells. Cancer Res..

[88] Hasegawa H., Komoda M., Yamada Y., Yonezawa S., Tsutsumida H., Nagai K., Atogami S., Tsuruda K., Osaka A., Sasaki D. (2011). Aberrant overexpression of membrane-associated mucin contributes to tumor progression in adult T-cell leukemia/lymphoma cells. Leuk. Lymphoma.

[89] Ishida T., Joh T., Uike N., Yamamoto K., Utsunomiya A., Yoshida S., Saburi Y., Miyamoto T., Takemoto S., Suzushima H. (2012). Defucosylated anti-CCR4 monoclonal antibody (KW-0761) for relapsed adult T-cell leukemia-lymphoma: A multicenter phase II study. J. Clin. Oncol..

[90] Tobinai K., Takahashi T., Akinaga S. (2012). Targeting chemokine receptor CCR4 in adult T-cell leukemia-lymphoma and other T-cell lymphomas. Curr. Hematol. Malig. Rep..

[91] Ishida T., Fujiwara H., Nosaka K., Taira N., Abe Y., Imaizumi Y., Moriuchi Y., Jo T., Ishizawa K., Tobinai K. (2016). Multicenter Phase II Study of Lenalidomide in Relapsed or Recurrent Adult T-Cell Leukemia/Lymphoma: ATLL-002. J. Clin. Oncol..

[92] Horwitz S., O’Connor O.A., Pro B., Trumper L., Iyer S., Advani R., Bartlett N.L., Christensen J.H., Morschhauser F., Domingo-Domenech E. (2022). The ECHELON-2 Trial: 5-year results of a randomized, phase III study of brentuximab vedotin with chemotherapy for CD30-positive peripheral T-cell lymphoma. Ann. Oncol..

[93] Yoshimitsu M., Ando K., Ishida T., Yoshida S., Choi I., Hidaka M., Takamatsu Y., Gillings M., Lee G.T., Onogi H. (2022). Oral histone deacetylase inhibitor HBI-8000 (tucidinostat) in Japanese patients with relapsed or refractory non-Hodgkin’s lymphoma: Phase I safety and efficacy. Jpn. J. Clin. Oncol..

[94] Izutsu K., Makita S., Nosaka K., Yoshimitsu M., Utsunomiya A., Kusumoto S., Morishima S., Tsukasaki K., Kawamata T., Ono T. (2023). An open-label, single-arm phase 2 trial of valemetostat for relapsed or refractory adult T-cell leukemia/lymphoma. Blood.

[95] Yamagishi M., Hori M., Fujikawa D., Ohsugi T., Honma D., Adachi N., Katano H., Hishima T., Kobayashi S., Nakano K. (2019). Targeting Excessive EZH1 and EZH2 Activities for Ab-normal Histone Methylation and Transcription Network in Malignant Lymphomas. Cell Rep..

[96] Cheong J.W., Chong S.Y., Kim J.Y., Eom J.I., Jeung H.K., Maeng H.Y., Lee S.T., Min Y.H. (2003). Induction of apoptosis by apicidin, a histone deacetylase inhibitor, via the activation of mitochondria-dependent caspase cascades in human Bcr-Abl-positive leukemia cells. Clin. Cancer Res..

[97] Laubach J.P., Moreau P., San-Miguel J.F., Richardson P.G. (2015). Panobinostat for the Treatment of Multiple Myeloma. Clin. Cancer Res..

[98] Morabito F., Voso M.T., Hohaus S., Gentile M., Vigna E., Recchia A.G., Iovino L., Benedetti E., Lo-Coco F., Galimberti S. (2016). Panobinostat for the treatment of acute myelogenous leukemia. Expert Opin. Investig. Drugs.

[99] Ikebe E., Kawaguchi A., Tezuka K., Taguchi S., Hirose S., Matsumoto T., Mitsui T., Senba K., Nishizono A., Hori M. (2013). Oral administration of an HSP90 inhibitor, 17-DMAG, intervenes tumor-cell infiltration into multiple organs and improves survival period for ATL model mice. Blood Cancer J..

[100] Ikebe E., Shimosaki S., Hasegawa H., Iha H., Tsukamoto Y., Wang Y., Sasaki D., Imaizumi Y., Miyazaki Y., Yanagihara K. (2022). TAS-116 (pimitespib), a heat shock protein 90 inhibitor, shows efficacy in preclinical models of adult T-cell leukemia. Cancer Sci..

[101] Taniguchi H., Hasegawa H., Sasaki D., Ando K., Sawayama Y., Imanishi D., Taguchi J., Imaizumi Y., Hata T., Tsukasaki K. (2014). Heat shock protein 90 inhibitor NVP-AUY922 exerts potent activity against adult T-cell leukemia-lymphoma cells. Cancer Sci..

[102] Sharp S.Y., Boxall K., Rowlands M., Prodromou C., Roe S.M., Maloney A., Powers M., Clarke P.A., Box G., Sanderson S. (2007). In vitro biological characterization of a novel, synthetic diaryl pyrazole resorcinol class of heat shock protein 90 inhibitors. Cancer Res..

[103] Doi T., Kurokawa Y., Sawaki A., Komatsu Y., Ozaka M., Takahashi T., Naito Y., Ohkubo S., Nishida T. (2019). Efficacy and safety of TAS-116, an oral inhibitor of heat shock protein 90, in patients with metastatic or unresectable gastrointestinal stromal tumour refractory to imatinib, sunitinib and regorafenib: A phase II, single-arm trial. Eur. J. Cancer.

[104] Xiao Y., Xu G., Cloyd J.M., Du S., Mao Y., Pawlik T.M. (2022). Predicting Novel Drug Candidates for Pancreatic Neuroendocrine Tumors via Gene Signature Comparison and Connectivity Mapping. J. Gastrointest. Surg..

[105] Katoh M., Nakagama H. (2014). FGF receptors: Cancer biology and therapeutics. Med. Res. Rev..

[106] Sampath D., Malik A., Plunkett W., Nowak B., Williams B., Burton M., Verstovsek S., Faderl S., Garcia-Manero G., List A.F. (2013). Phase I clinical, pharmacokinetic, and pharmacodynamic study of the Akt-inhibitor triciribine phosphate monohydrate in patients with advanced hematologic malignancies. Leuk. Res..

[107] Ranieri R., Pianigiani G., Sciabolacci S., Perriello V.M., Marra A., Cardinali V., Pierangeli S., Milano F., Gionfriddo I., Brunetti L. (2022). Current status and future perspectives in targeted therapy of NPM1-mutated AML. Leukemia.

[108] Inchiosa M.A. (2023). Further investigation of the potential anti-neoplastic, anti-inflammatory and immunomodulatory actions of phenoxybenzamine using the Broad Institute CLUE platform. J. Pharm. Pharmacol. Res..

[109] Grimsby J., Sarabu R., Corbett W.L., Haynes N.E., Bizzarro F.T., Coffey J.W., Guertin K.R., Hilliard D.W., Kester R.F., Mahaney P.E. (2003). Allosteric activators of glucokinase: Potential role in diabetes therapy. Science.

[110] Diaz O., Vidalain P.O., Ramiere C., Lotteau V., Perrin-Cocon L. (2022). What role for cellular metabolism in the control of hepatitis viruses?. Front. Immunol..

[111] Grignano E., Birsen R., Chapuis N., Bouscary D. (2020). From Iron Chelation to Overload as a Therapeutic Strategy to Induce Ferroptosis in Leukemic Cells. Front. Oncol..

[112] Yu Y., Xie Y., Cao L., Yang L., Yang M., Lotze M.T., Zeh H.J., Kang R., Tang D. (2015). The ferroptosis inducer erastin enhances sensitivity of acute myeloid leukemia cells to chemotherapeutic agents. Mol. Cell Oncol..

[113] Mynott R.L., Habib A., Best O.G., Wallington-Gates C.T. (2023). Ferroptosis in Haematological Malignancies and Associated Therapeutic Nanotechnologies. Int. J. Mol. Sci..

