# Supplementary material for: The Novel Link between Gene Expression Profiles of Adult T-Cell Leukemia/Lymphoma Patients’ Peripheral Blood Lymphocytes and Ferroptosis Susceptibility
Source: Genes (Basel). 2023 Oct 27;14(11):2005. doi: 10.3390/genes14112005 (PMC10671613; doi:10.3390/genes14112005)
Supplement: Supplementary file 1 [file genes-14-02005-s001.zip › 231019_3_Genes-2648278_Supplementary_files/Supplementary figure S1-S3.pdf]

Supplement Figure 1: The enrichment pathway analysis of ferroptosis-related DEGs

A

B

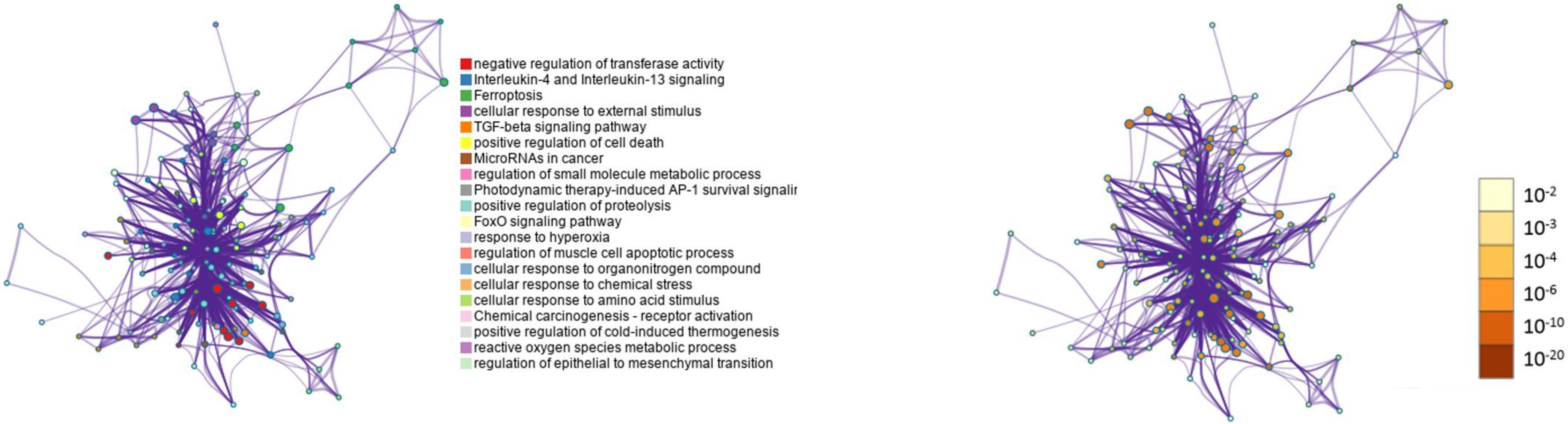

Supplementary Figure 2: The enrichment pathway analysis of autophagy-related DEGs

A

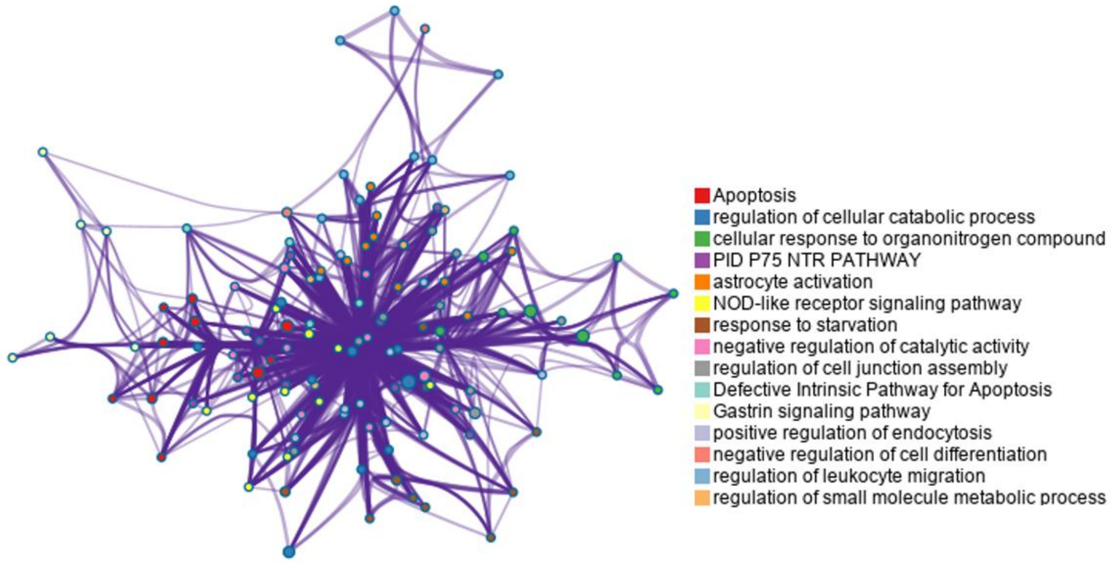

B

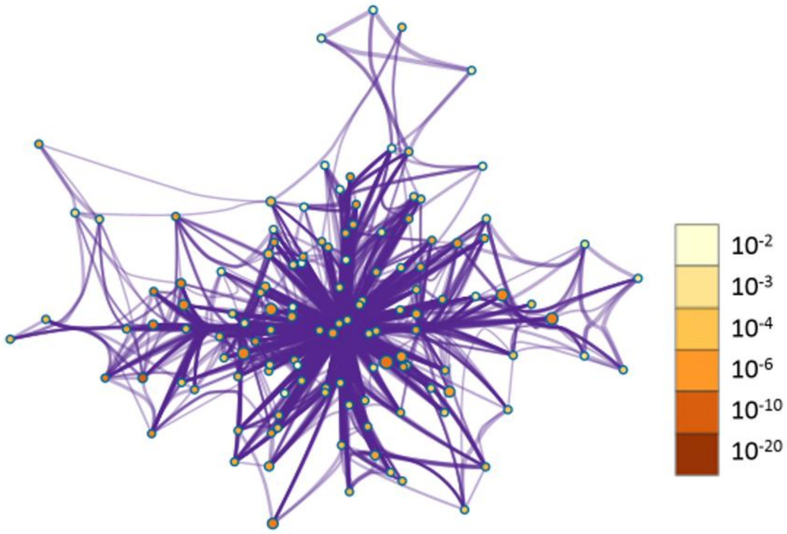

Supplementary Figure 3 The network of Target genes- TF and -miRNA

A

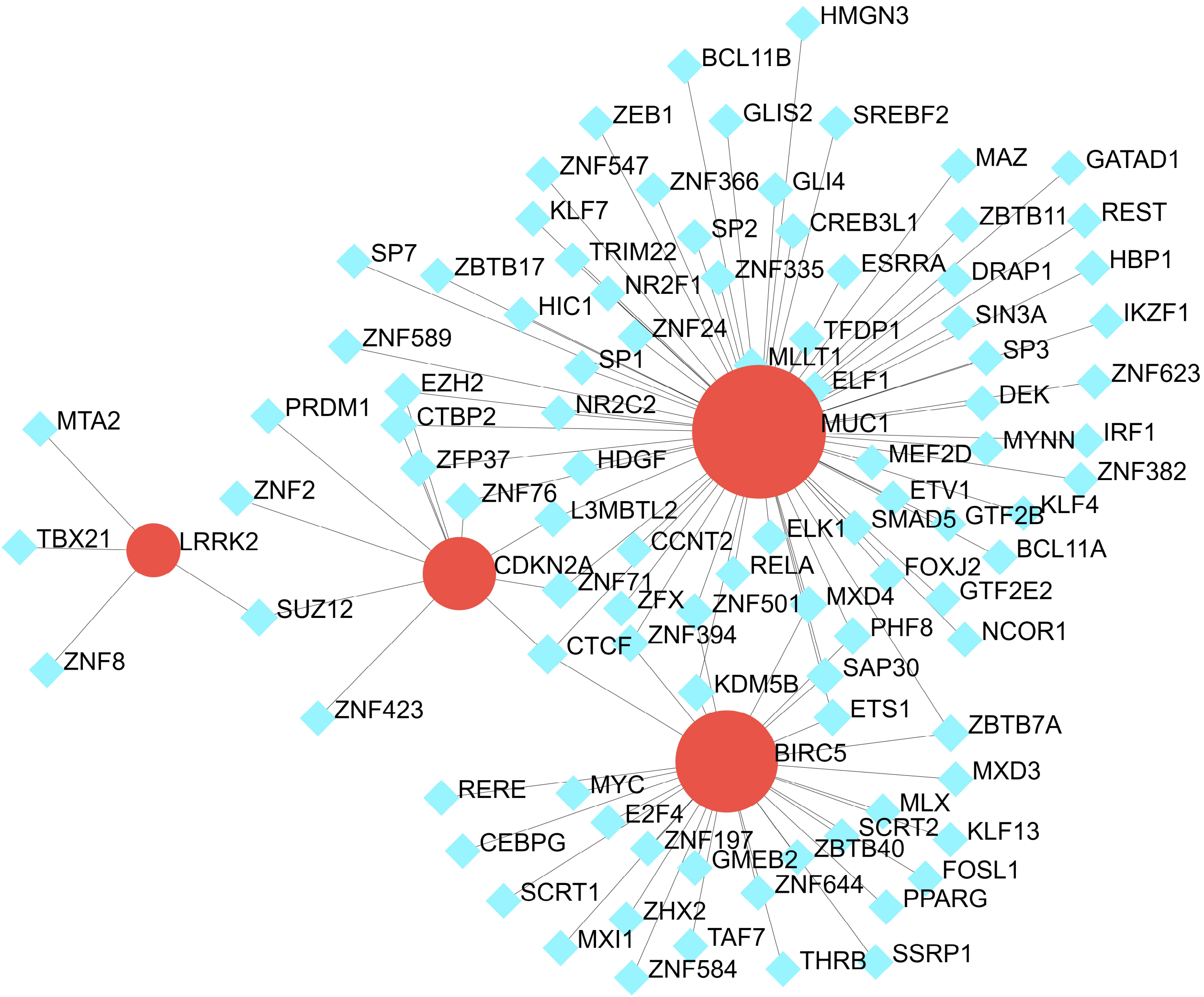

B

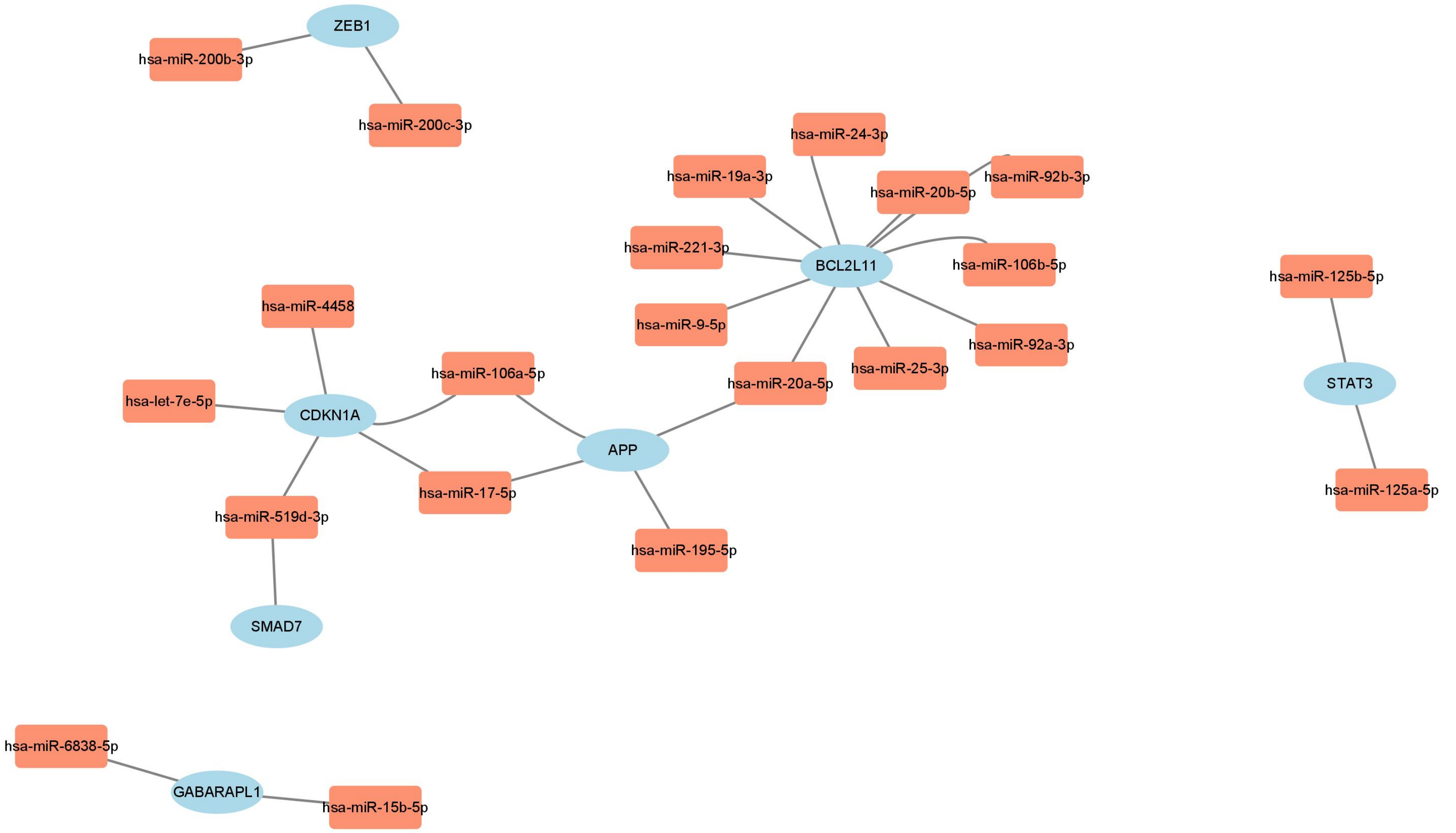
ZEB1
hsa-miR-200b-3p
hsa-miR-200c-3p
hsa-miR-24-3p
hsa-miR-19a-3p
hsa-miR-92b-3p
hsa-miR-20b-5p
hsa-miR-221-3p
BCL2L11
hsa-miR-106b-5p
hsa-miR-125b-5p
hsa-miR-4458
hsa-miR-9-5p
hsa-miR-92a-3p
hsa-miR-106a-5p
hsa-miR-25-3p
hsa-let-7e-5p
CDKN1A
hsa-miR-20a-5p
STAT3
APP
hsa-miR-17-5p
hsa-miR-125a-5p
hsa-miR-519d-3p
hsa-miR-195-5p
SMAD7
hsa-miR-6838-5p
GABARAPL1
hsa-miR-15b-5p
