# Supplementary material for: The Novel Link between Gene Expression Profiles of Adult T-Cell Leukemia/Lymphoma Patients’ Peripheral Blood Lymphocytes and Ferroptosis Susceptibility
Source: Genes (Basel). 2023 Oct 27;14(11):2005. doi: 10.3390/genes14112005 (PMC10671613; doi:10.3390/genes14112005)
Supplement: Supplementary file 1 [file genes-14-02005-s001.zip › 231019_3_Genes-2648278_Supplementary_files/Supplementary Table S1-S6.pdf]

Supplementary Table S1. Ferroptosis-related differentially expressed genes of ATL.

genesybol	adj.P.Val	logFC	Gene Title	id
HBA1	1.29E-18	6.4372	hemoglobin, alpha 1	35676
NEDD4L	2.16E-07	3.2987	neural precursor cell expressed, developmentally down-regulated 4-like"	16818
TP63	5.12E-05	2.5939	tumor protein p63	28920
VLDLR	6.82E-07	2.5599	very low density lipoprotein receptor	7516
TF	1.89E-14	2.4631	transferrin	792
WWTR1	1.37E-07	2.1082	WW domain containing transcription regulator 1	12258
CAV1	2.23E-03	2.0759	caveolin 1, caveolae protein, 22kDa"	33242
CREB5	5.15E-06	2.0431	cAMP responsive element binding protein 5	35433
RRM2	1.96E-04	1.9609	ribonucleotide reductase M2 polypeptide	21855
GPT2	4.76E-05	1.8667	glutamic pyruvate transaminase (alanine aminotransferase) 2	39644
CDC25A	7.30E-05	1.8540	cell division cycle 25 homolog A (S. pombe)	1097
CAPG	9.41E-09	1.8137	capping protein (actin filament), gelsolin-like"	18229
MGST1	3.56E-04	1.6725	microsomal glutathione S-transferase 1	6390
PEX6	3.70E-08	1.6342	peroxisomal biogenesis factor 6	9186
CDKN2A	1.06E-05	1.5771	cyclin-dependent kinase inhibitor 2A (melanoma, p16, inhibits CDK4)"	1687
TRIB2	5.89E-07	1.5699	tribbles homolog 2 (Drosophila)	22976
MAPKAP1	1.40E-13	1.5590	mitogen-activated protein kinase associated protein 1	24217
MT1G	3.01E-06	1.5454	metallothionein 1G	22896
PDK4	2.52E-03	1.5406	pyruvate dehydrogenase kinase, isozyme 4"	42318
MUC1	4.65E-06	1.5266	mucin 1, cell surface associated"	28892
STEAP3	7.89E-05	1.5105	STEAP family member 3	6929
MAPK8	8.19E-09	-1.5254	mitogen-activated protein kinase 8	30035
SLC7A5	4.83E-06	-1.5717	solute carrier family 7 (cationic amino acid transporter, y+ system), member 5"	2090
SCD	2.41E-07	-1.6331	stearoyl-CoA desaturase (delta-9-desaturase)	41666
PARP11	5.61E-10	-1.6501	poly (ADP-ribose) polymerase family, member 11"	26888
STAT3	1.38E-09	-1.7027	signal transducer and activator of transcription 3 (acute-phase response factor)	27157
SLC7A11	5.83E-08	-1.7518	solute carrier family 7, (cationic amino	32586

			acid transporter, y+ system) member 11"	
ZFP36	2.06E-11	-1.7783	zinc finger protein 36, C3H type, homolog (mouse)"	2800
ZEB1	3.64E-07	-1.8059	zinc finger E-box binding homeobox 1	18088
AKR1C3	3.02E-04	-1.8111	aldo-keto reductase family 1, member C3 (3-alpha hydroxysteroid dehydrogenase, type II)"	42774
BNIP3	1.14E-10	-1.8593	BCL2/adenovirus E1B 19kDa interacting protein 3	29331
IFNG	2.08E-04	-1.8867	interferon, gamma"	16569
LGMN	3.06E-05	-1.9617	legumain	39661
GABARAPL1	6.59E-09	-1.9746	GABA(A) receptor-associated protein like 1	6174
SOCS1	3.54E-12	-2.0723	suppressor of cytokine signaling 1	41081
SETD1B	3.29E-19	-2.1030	SET domain containing 1B	39229
RBMS1	1.85E-14	-2.1612	RNA binding motif, single stranded interacting protein 1"	11523
ENPP2	6.20E-06	-2.1921	ectonucleotide pyrophosphatase/phosphodiesterase 2	24072
DPP4	1.48E-14	-2.3504	dipeptidyl-peptidase 4	12264
USP35	6.58E-28	-2.4351	ubiquitin specific peptidase 35	19531
CDKN1A	2.48E-15	-2.5308	cyclin-dependent kinase inhibitor 1A (p21, Cip1)"	15513
SLC40A1	3.51E-08	-2.7593	solute carrier family 40 (iron-regulated transporter), member 1"	19903
LCN2	6.04E-07	-2.7757	lipocalin 2	33122
IL1B	3.82E-06	-2.7949	interleukin 1, beta"	17980
BEX1	3.17E-14	-3.6296	brain expressed, X-linked 1	38982
SMAD7	3.03E-16	-3.9545	SMAD family member 7	351

Supplementary Table S2. The ferroptosis-related differentially expressed genes were divided into ferroptosis suppressor, driver, unclassified and marker

suppressor	driver	unclassified	marker
BEX1	SMAD7	HBA1	SLC40A1
NEDD4L	IL1B	VLDLR	TF
LCN2	TF	TF	
SLC40A1	DPP4	SETD1B	
TP63	WWTR1	RRM2	
CDKN1A	SOCS1	GPT2	
TF	GABARAPL1	BNIP3	
USP35	LGMN	CAPG	
ENPP2	IFNG	SLC7A11	
RBMS1	ZEB1	SLC7A5	
CAV1	SLC7A11	STEAP3	
CREB5	PEX6		
RRM2	CDKN2A		
CDC25A	MAPK8		
AKR1C3			
ZFP36			
SLC7A11			
STAT3			
MGST1			
PARP11			
SCD			
TRIB2			
MAPKAP1			
MT1G			
PDK4			
MUC1			

Supplementary Table S3. Autophagy-related differentially expressed genes of ATL.

genesymbol	adj.P.Val	logFC	Gene Title	id
TP63	5.12344E-05	2.5939	tumor protein p63	28920
LRRK2	4.87445E-06	2.2772	leucine-rich repeat kinase 2	20217
TP53INP1	1.04677E-11	1.9438	tumor protein p53 inducible nuclear protein 1	36711
CYB5A	3.37088E-05	1.7382	cytochrome b5 type A (microsomal)	44466
UBASH3B	1.38957E-07	1.5932	ubiquitin associated and SH3 domain containing, B	36160
TBC1D5	3.43742E-11	1.5848	TBC1 domain family, member 5"	39178
CDKN2A	1.06318E-05	1.5771	cyclin-dependent kinase inhibitor 2A (melanoma, p16, inhibits CDK4)"	1687
PDK4	0.002524317	1.5406	pyruvate dehydrogenase kinase, isozyme 4"	42318
BIRC5	0.002498051	1.5288	baculoviral IAP repeat-containing 5	28938
MAPK8	8.18775E-09	-1.5254	mitogen-activated protein kinase 8	30035
EEF2K	3.30867E-08	-1.5688	eukaryotic elongation factor-2 kinase	21439
GPR18	1.63242E-07	-1.7436	G protein-coupled receptor 18	28002
SNAI1	3.09973E-07	-1.7720	snail homolog 1 (Drosophila)	1390
BIRC2	9.04297E-27	-1.9537	baculoviral IAP repeat-containing 2	17712
GABARAPL1	6.5943E-09	-1.9746	GABA(A) receptor-associated protein like 1	6174
TNF	1.95885E-06	-2.0012	tumor necrosis factor (TNF superfamily, member 2)"	13025
APP	2.62527E-06	-2.0422	amyloid beta (A4) precursor protein	20175
SIK2	1.14839E-11	-2.3187	salt-inducible kinase 2	15147
MAPT	2.0207E-14	-2.5343	microtubule-associated protein tau	20433
BCL2L11	1.94785E-10	-2.5709	BCL2-like 11 (apoptosis facilitator)	32463
LZTS1	1.30016E-12	-3.0211	leucine zipper, putative tumor suppressor 1"	32127
LAPTM4B	2.64801E-13	-3.2081	lysosomal protein transmembrane 4 beta	22671
CAMP	1.13546E-06	-3.3231	cathelicidin antimicrobial peptide	26579
TBC1D4	9.798E-15	-3.8369	TBC1 domain family, member 4	4777
CXCR3	2.57717E-19	-3.8766	chemokine (C-X-C motif) receptor 3	23155
MID2	3.32244E-19	-4.3439	midline 2	23728

Supplementary Table S4. GO/KEGG enrichment analysis for Ferroptosis-related

Ontology	ID	Description	p.adjust	Count	zscore
BP	GO:0051348	negative regulation of transferase activity	2.42636E-05	9	-0.333333333
	GO:0071496	cellular response to external stimulus	0.001053878	7	-1.889822365
	GO:0062197	cellular response to chemical stress	0.001518472	7	-0.377964473
	GO:0042326	negative regulation of phosphorylation	2.42636E-05	10	-0.632455532
	GO:0030856	regulation of epithelial cell differentiation	4.16383E-05	7	-0.377964473
	GO:0001933	negative regulation of protein phosphorylation	4.16383E-05	9	-0.333333333
	GO:0010038	response to metal ion	4.5611E-05	9	-0.333333333
	GO:0006826	iron ion transport	8.5108E-05	5	-0.447213595
	GO:0050863	regulation of T cell activation	0.001598328	7	-1.889822365
CC	GO:0045177	apical part of cell	0.064255189	6	-0.816496581
MF	GO:0004860	protein kinase inhibitor activity	0.002977318	4	0
	GO:0019210	kinase inhibitor activity	0.002977318	4	0
	GO:0005506	iron ion binding	0.002977318	5	0.447213595
	GO:0008199	ferric iron binding	0.017642478	2	1.414213562
	GO:0004861	cyclin-dependent protein serine/threonine kinase inhibitor activity	0.017642478	2	0
	GO:0061629	RNA polymerase II-specific DNA-binding transcription factor binding	0.053844353	5	-0.447213595
KEGG	hsa04216	Ferroptosis	0.007337061	4	0
	hsa04068	FoxO signaling pathway	0.017693871	5	-2.236067977
	hsa05206	MicroRNAs in cancer	0.017693871	7	-0.377964473
	hsa04115	p53 signaling pathway	0.017693871	4	1
	hsa05212	Pancreatic cancer	0.017693871	4	-1
	hsa05418	Fluid shear stress and atherosclerosis	0.017693871	5	-0.447213595
	hsa04657	IL-17 signaling pathway	0.023030109	4	-2
	hsa04659	Th17 cell differentiation	0.029027441	4	-2
	hsa04066	HIF-1 signaling pathway	0.029027441	4	-1
	hsa05166	Human T-cell leukemia virus 1 infection	0.041852069	5	-0.447213595

Supplementary Table S5. GO/KEGG enrichment analysis for Autophagy-related

ONTOLOGY	ID	Description	p.adjust	Count	zscore
BP	GO:0042594	response to starvation	0.000158625	6	0
BP	GO:0048143	astrocyte activation	0.000480446	3	-1.732050808
BP	GO:0031331	positive regulation of cellular catabolic process	0.001812582	6	-0.816496581
BP	GO:0010506	regulation of autophagy	0.000158625	7	-1.133893419
BP	GO:0043281	regulation of cysteine-type endopeptidase activity involved in apoptotic process	0.000158625	6	-0.816496581
BP	GO:1901653	cellular response to peptide	0.000158625	7	-1.889822365
BP	GO:0001774	microglial cell activation	0.000158625	4	-1
BP	GO:2000116	regulation of cysteine-type endopeptidase activity	0.000158625	6	-0.816496581
BP	GO:0002269	leukocyte activation involved in inflammatory response	0.000158625	4	-1
MF	GO:0015631	tubulin binding	9.10484E-05	7	-1.133893419
MF	GO:0008017	microtubule binding	0.00012198	6	-0.816496581
MF	GO:0004857	enzyme inhibitor activity	0.008716538	5	0.447213595
MF	GO:0043027	cysteine-type endopeptidase inhibitor activity involved in apoptotic process	0.012089896	2	0
MF	GO:0051087	chaperone binding	0.012089896	3	-0.577350269
MF	GO:0043028	cysteine-type endopeptidase regulator activity involved in apoptotic process	0.033290429	2	0
CC	GO:0005776	autophagosome	0.001729195	4	1
CC	GO:0045121	membrane raft	0.0018998	5	-1.341640786
CC	GO:0098857	membrane microdomain	0.0018998	5	-1.341640786
KEGG	hsa04210	Apoptosis	0.000775323	5	-1.341640786
KEGG	hsa04621	NOD-like receptor signaling pathway	0.002229251	5	-2.236067977
KEGG	hsa04215	Apoptosis - multiple species	9.76225E-05	4	-1
KEGG	hsa01524	Platinum drug resistance	0.020240912	3	0.577350269
KEGG	hsa05210	Colorectal cancer	0.026139473	3	-0.577350269
KEGG	hsa04931	Insulin resistance	0.03501896	3	-1.732050808
KEGG	hsa04668	TNF signaling pathway	0.03501896	3	-1.732050808
KEGG	hsa05145	Toxoplasmosis	0.03501896	3	-1.732050808
KEGG	hsa04068	FoxO signaling pathway	0.048627811	3	-1.732050808

Supplementary Table S6. GO/KEGG enrichment analysis for Hub genes of Ferroptosis- and Autophagy-related DEGs.

ONTOLOGY	ID	Description	p.adjust	Count	zscore
BP	GO:0061900	glial cell activation	1.17856E-08	6	-1.632993162
BP	GO:0045862	positive regulation of proteolysis	1.17856E-08	9	-2.333333333
BP	GO:0150076	neuroinflammatory response	2.2193E-08	6	-1.632993162
BP	GO:0045936	negative regulation of phosphate metabolic process	2.2193E-08	9	-1.666666667
BP	GO:0048143	astrocyte activation	1.17856E-08	5	-2.236067977
BP	GO:0002269	leukocyte activation involved in inflammatory response	1.72726E-07	5	-1.341640786
BP	GO:0042326	negative regulation of phosphorylation	1.72726E-07	8	-1.414213562
BP	GO:0032755	positive regulation of interleukin-6 production	3.67117E-06	5	-2.236067977
CC	GO:0045121	membrane raft	0.000544419	5	-1.341640786
CC	GO:0098857	membrane microdomain	0.000544419	5	-1.341640786
CC	GO:0030426	growth cone	0.010929308	3	-0.577350269
CC	GO:0097386	glial cell projection	0.010929308	2	-1.414213562
CC	GO:0030427	site of polarized growth	0.010929308	3	-0.577350269
KEGG	hsa04215	Apoptosis - multiple species	3.65445E-05	4	-1
KEGG	hsa04217	Necroptosis	3.65445E-05	6	-2.449489743
KEGG	hsa05145	Toxoplasmosis	0.000114108	5	-2.236067977
KEGG	hsa04068	FoxO signaling pathway	0.00017832	5	-2.236067977
KEGG	hsa04210	Apoptosis	0.00017832	5	-1.341640786
KEGG	hsa05321	Inflammatory bowel disease	0.000190727	4	-2
KEGG	hsa01524	Platinum drug resistance	0.000237581	4	0

Supplementary Table S7. GO/KEGG enrichment analysis for Hub genes of Ferroptosis- and Autophagy-related DEGs.

ONTOLOGY	ID	Description	p.adjust	Count	zscore
MF	GO:0004857	enzyme inhibitor activity	0.000162684	6	0
MF	GO:0015631	tubulin binding	0.001488279	5	-0.447213595
MF	GO:0005126	cytokine receptor binding	0.002961663	4	-2
MF	GO:0008017	microtubule binding	0.002961663	4	0
MF	GO:0051087	chaperone binding	0.002961663	3	-0.577350269
MF	GO:0019903	protein phosphatase binding	0.005940815	3	-1.732050808
MF	GO:0004866	endopeptidase inhibitor activity	0.007469973	3	-0.577350269
MF	GO:0030414	peptidase inhibitor activity	0.007469973	3	-0.577350269
MF	GO:0019902	phosphatase binding	0.007469973	3	-1.732050808
MF	GO:0061135	endopeptidase regulator activity	0.007469973	3	-0.577350269
MF	GO:0004861	cyclin-dependent protein serine/threonine kinase inhibitor activity	0.002468614	2	0
MF	GO:0043027	cysteine-type endopeptidase inhibitor activity involved in apoptotic process	0.00368153	2	0
MF	GO:0030291	protein serine/threonine kinase inhibitor activity	0.006503378	2	0
MF	GO:0043028	cysteine-type endopeptidase regulator activity involved in apoptotic process	0.007469973	2	0
MF	GO:0016538	cyclin-dependent protein serine/threonine kinase regulator activity	0.008964342	2	0
