# Supplementary figures and images for: The Novel Link between Gene Expression Profiles of Adult T-Cell Leukemia/Lymphoma Patients’ Peripheral Blood Lymphocytes and Ferroptosis Susceptibility
Source: Genes (Basel). 2023 Oct 27;14(11):2005. doi: 10.3390/genes14112005 (PMC10671613; doi:10.3390/genes14112005)

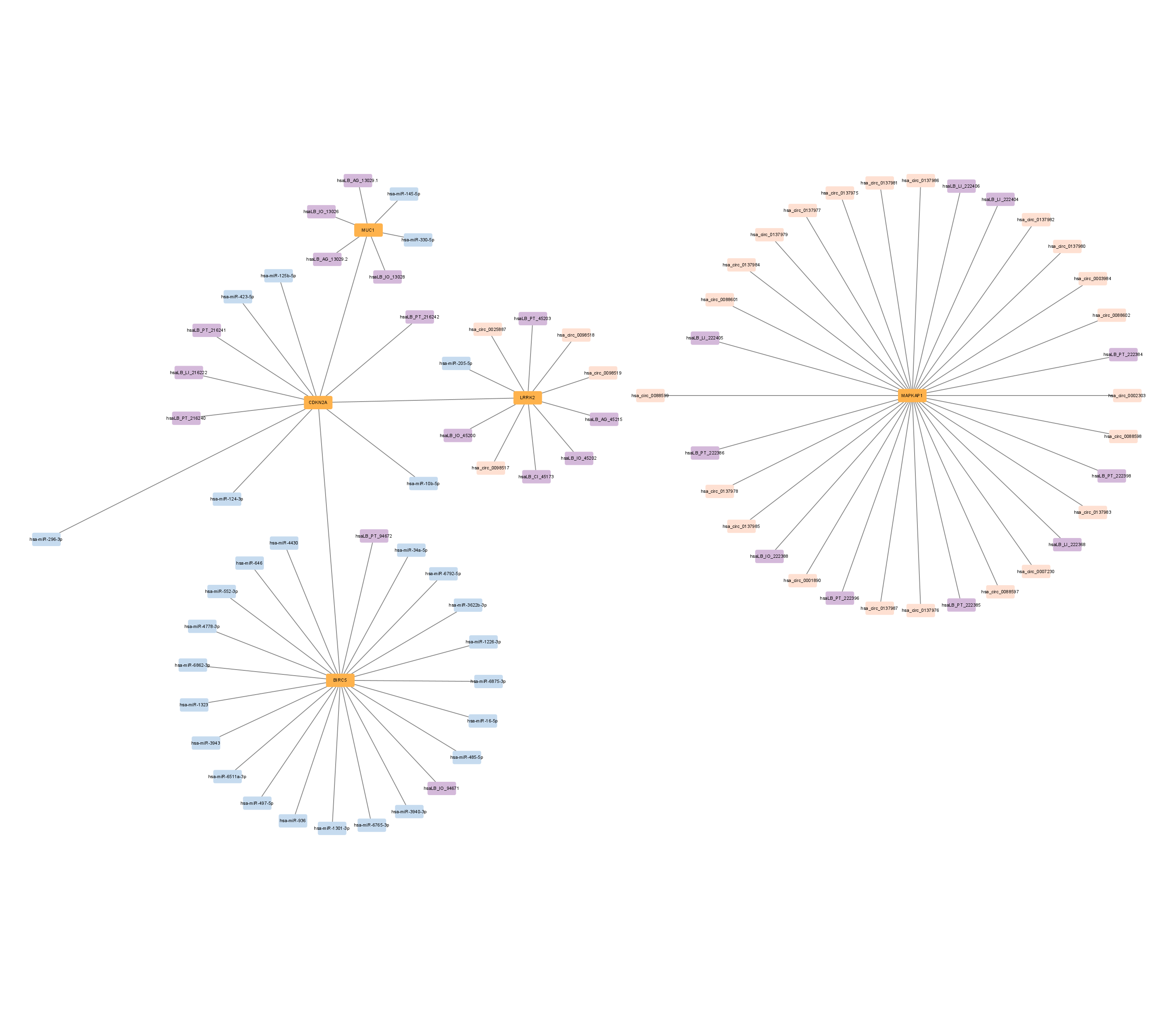

Supplement: Supplementary file 1 [file genes-14-02005-s001.zip › 231019_3_Genes-2648278_Supplementary_files/Supplementary Figure S4.png]
